# Integrated analysis of lncRNAs and mRNAs by RNA-Seq in secondary hair follicle development and cycling (anagen, catagen and telogen) of Jiangnan cashmere goat (*Capra hircus)*

**DOI:** 10.1186/s12917-022-03253-0

**Published:** 2022-05-06

**Authors:** Cuiling Wu, Chongkai Qin, Xuefeng Fu, Xixia Huang, Kechuan Tian

**Affiliations:** 1 College of Animal Science, Xinjiang Agricultural University, Urumqi, 830052 China; 2 Institute of Animal Science and Veterinary Medicine, Shandong Academy of Agricultural Sciences, Jinan, 250100 China; 3 Key Laboratory of Genetics Breeding and Reproduction of Xinjiang Wool sheep & Cashmere-goat, Institute of Animal Science, Xinjiang Academy of Animal Sciences, Urumqi, 830011 China; 4 Xinjiang Aksu Prefecture Animal Husbandry Technology Extension Center, Aksu, 843000 China

**Keywords:** Goat hair follicles, Signaling pathways, Differentially expressed genes, Photoperiodism

## Abstract

**Background:**

Among the world’s finest natural fiber composites is derived from the secondary hair follicles (SHFs) of cashmere goats yield one of the world's best natural fibres. Their development and cycling are characterized by photoperiodism with diverse, well-orchestrated stimulatory and inhibitory signals. Long non-coding RNA (lncRNAs) and mRNAs play important roles in hair follicle (HF) development. However, not many studies have explored their specific functions in cashmere development and cycling. This study detected mRNAs and lncRNAs with their candidate genes and related pathways in SHF development and cycling of cashmere goat. We utilized RNA sequencing (RNA-Seq) and bioinformatics analysis on lncRNA and mRNA expressions in goat hair follicles to discover candidate genes and metabolic pathways that could affect development and cycling (anagen, catagen, and telogen).

**Results:**

We identified 228 differentially expressed (DE) mRNAs and 256 DE lncRNA. For mRNAs, catagen and anagen had 16 upregulated and 35 downregulated DEGs, catagen and telogen had 18 upregulated and 9 downregulated DEGs and telogen and anagen had 52 upregulated and 98 downregulated DEGs. LncRNA witnessed 22 upregulated and 39 downregulated DEGs for catagen and anagen, 36 upregulated and 29 downregulated DEGs for catagen and telogen as well as 66 upregulated and 97 downregulated DEGs for telogen and anagen. Several key genes, including *MSTRG.5451.2, MSTRG.45465.3, MSTRG.11609.2, CHST1, SH3BP4, CDKN1A, GAREM1, GSK-3β, DEFB103A KRTAP9–2, YAP1, S100A7A, FA2H, LOC102190037, LOC102179090, LOC102173866, KRT2, KRT39, FAM167A, FAT4* and *EGFL6* were shown to be potentially important in hair follicle development and cycling. They were related to, WNT/β-catenin, mTORC1, ERK/MAPK, Hedgehog, TGFβ, NFkB/p38MAPK, caspase-1, and interleukin (IL)-1a signaling pathways.

**Conclusion:**

This work adds to existing understanding of the regulation of HF development and cycling in cashmere goats via lncRNAs and mRNAs. It also serves as theoretical foundation for future SHF research in cashmere goats.

## Background

In the 1970s, Xinjiang became China’s first international cashmere market, and it has since become a cashmere powerhouse. China provides a distinct husbandry resource called cashmere. It produces 12,000 tons of cashmere per year, accounting for more than 70% of global output [1, 2]. Cashmere goat fleece have two coats; wool is made by primary hair follicles (PHF) and cashmere is produced by secondary hair follicles (SHF) [3–5]. Cashmere is a delicate (dehaired) downy undercoat shed by goats that lies beneath the outer thick hair shaft to provide an adaptability to cold [1, 6]. Primary and secondary follicles differ particularly in terms of morphogenesis, cycle development, and positioning of hair follicles. The SHF does not have sebaceous gland like PHF [4] and follicle diameter and dermal papilla (DP) size are larger in PHF than the SHF [7]. Jiangnan cashmere goat is widely known for producing fine and silky hair fiber with great quality, economic value and high yield from SHF. Its extraordinary fiber features are related to its genetics and breeding qualities [8]. Cashmere is eight times warmer and just as soft as sheep wool, providing excellent insulation [9]. It remains one of the most valuable natural products in sales. While current output is dropping, global demand for cashmere in the textile and fabric business is increasing [10, 11]. Consequently, breeding high-quality and super-fineness cashmere has become a pressing issue in their breeding sector. SHF developmental characteristics influence cashmere yield and quality [12]. As a result, studies on the molecular mechanisms regulating cashmere goat HF development have become a research focus. As cashmere development is characterized by photoperiodic dependency, which occurs with cyclic regularity of SHF in quick growth (anagen), slow regression (catagen), and rest stage (telogen) [3, 6, 13, 14], exploring the main genes, signaling pathways and the complex regulatory mechanisms that enable their formation and development are important and must be investigated. Several key pathways involved in cashmere goat HF development and cycling include tumor necrosis factor (TNF), MAPK signaling pathway, WNT pathway, fibroblast growth factor (FGF) family, bone morphogenetic protein (BMP) family, transforming growth factor (TGF) family, Sonic hedgehog (SHH) conduction pathway and NOTCH signal transduction pathway [3, 15–18]. However, not many signaling pathways that inhibit or enhance SHF development have been extensively investigated, and certain pathways are yet to be uncovered and comprehended. Current studies looking beyond protein-coding genes have concluded that non-coding RNA (ncRNA) such as microRNA (miRNA), natural antisense transcripts (NAT) and long non-coding RNA (lncRNA) may be more specific as biomarkers for certain applications than protein coding genes [13]. Among these ncRNAs, lncRNAs are abundant and also play crucial roles in cell development, cycle, proliferation and differentiation [18–20]. They perform functions, including transcriptional activation, protein coding gene silencing and mRNA or miRNA interaction to regulate their actions [21, 22]. While the understanding of the diverse regulatory mechanisms of ncRNAs is far from thorough, new studies have shown specific lncRNA pathways in HF morphogenesis in goats and sheeps [6, 18]. A previous study in the expression profiles of lncRNAs and mRNAs throughout sheep fetal and postnatal hair follicle demonstrated that the integrative function of lncRNA and their target genes regulate HF development [23]. Another study used strand specific RNA sequencing (ssRNA-Seq) to ascertain the roles of lncRNAs and mRNAs in sheep skin at the on-set of SHF development and it was concluded that the key differentially expressed genes (DEGs) and lncRNAs may affect the molecular mechanisms involved in HF initiation [18]. Again, a study of the regulatory relationships of lncRNAs, miRNAs and mRNAs in the goat HF cycle was performed mainly in the catagen and anagen phases. It was revealed that lncRNAs and miRNAs directly correlate in HF cycling and catagen inducer factors transforming growth factor beta 1 *(TGFβ1)* and brain-derived neurotrophic factor *(BDNF)* were regulated by miR-873 and lnc108635596 [24]. A comprehensive analysis of DE mRNAs and DE lncRNAs between Inner Mongolia cashmere goats and Liaoning cashmere goats revealed that lncRNA *XLOC_008679* and *KRT35* target gene could be candidates for regulating cashmere fineness [5]. However, the roles of lncRNAs and mRNAs in cashmere development and cycling have not been thoroughly explored.

In this current study, the SHF of Jiangnan cashmere goat serve as an ideal model for investigating hair biology because of the circannual rhythmicity and synchronized growth of the fiber. We used RNA sequencing (RNA-seq) to investigate the profiles of lncRNAs and mRNAs in Jiangnan cashmere goat SHF in anagen, catagen and telogen stages as well as explore the potential interactions with regulatory networks of the related DEGs. This study will add more to our understanding of lncRNA and mRNAs in HF morphogenesis and contribute to the improvement of cashmere goat breeding. It will also serve as beneficial reference for Jiangnan cashmere goat genetic breeding as well as contribute to the annotation of the goat genome.

## Materials and methods

### Animal ethics

All animal experiments were carried out in strict accordance with the instructions provided by the Animal Care and Use Committee of Xinjiang Academy of Animal Science (Approval number 2020008). Effective procedures were implemented to reduce pain and distress; overall health, zoonotic infections, and pathogenic microbial infectious diseases were all thoroughly controlled and monitored [6, 25].

### Experimental animal and sample collection

Experimental cashmere goats with high production features were collected from breeding farm in breeding center of Wenshu County, Aksu Prefecture, Xinjiang Province. The cashmere goats were fed in accordance with the standards of Xinjiang. Three female adults were chosen (two-year-old Jiangnan cashmere goat with a coefficient of relationship of < 0.125). Scapular skin tissues were collected from the goats in vivo. From each piece of skin tissue, approximately two cm^2^ and was immediately frozen by liquid nitrogen and stored at − 80 °C until further analysis [6].

### Total RNA isolation, library preparation and sequencing

Total RNA was isolated and purified (Invitrogen, USA) and lncRNA and mRNA libraries were prepared as described before [6, 26]. The integrity of total RNA was assessed using Agilent 2100 Bioanalyzer (Agilent Technologies Inc., USA) and sample with RNA Integrity number (RIN) values higher than 7.0 were used for sequencing. One microgram of RNA was used as input material for the RNA sample preparation. Subsequently, 9 cDNA libraries for the three developmental stages were constructed using rRNA-depleted RNA with the NEBNext® Ultra™ Directional RNA Library Prep Kit for Illumina® (NEB, USA) following the manufacturer’s instructions and each sample type contained three biological replicates. Libraries were sequenced using Illumina Hiseq 4000 platform (LC Bio, Hangzhou, Zhejiang, China) and 150-bp paired-end reads were generated. The raw data were assessed for quality using FastQC software (http://www.bioinformatics.babraham.ac.uk/projects/fastqc/). Clean reads were assessed by removing adapters and poly-*N* > 10%, and low-quality reads in the raw data using the Fastx toolkit. Clean reads were mapped to goat reference sequences (*Capra hircus ARS1.93*) using Hisat [6]. Reference genome and annotation files were obtained from the Ensembl browser (https://www.ncbi.nlm.nih.gov/genome/?term=capra+hircus+ars1). Cufflinks software was used to assemble transcripts [27].

### Coding potential and conserved analysis of lncRNAs

Based on the Cufflinks splicing results, lncRNA was chosen as final candidate for further analysis. The following parameters were used specifically; transcripts with transcript length ≥ 200 bp and exon number ≥ 2 were selected. The candidate transcripts were screened, using the class-code information from Cuffcompare. Cufflinks determined the transcript with coverage > 3 in at least one sample. Finally, we used coding potential analysis method, Coding-Non-Coding Index (CNCI), Coding Potential Calculator (CPC), PFAM and CPAT to choose the valid transcripts [6].

### Analysis of differentially expressed (DE) lncRNAs and mRNAs

Gene abundance was expressed as fragment per kilobase of exons per million reads (FPKM) and lncRNAs and mRNAs in each sample was calculated using Stringtie software [28]. TMM algorithm was used for normalization. The R package edgeR [29] was used for differential expression analysis of mRNA and lncRNA. DE RNAs with *p*-values < 0.05 and |log2FoldChange| ≥ 1 served as thresholds to assess the statistical significance of mRNA and lncRNA expression differences.

### Enrichment analysis and protein-protein interaction (PPI) of DE lncRNAs and mRNAs

GOseq R package was used for Gene ontology (GO) enrichment analysis and Kyoto Encyclopedia of Genes and Genomes (KEGG) pathway enrichment analyses. The Benjamini-Hochberg (BH) method [30] was used to adjust the significant *p*-values. GO terms and pathways with *p*-values < 0.05 were considered significantly enriched. The GO database was used to conduct functional annotations on DE transcripts, which were classified into three categories: biological processes (BPs), molecular function (MF), and cellular component (CC). Candidate genes encoded functional PPI network and were analyzed with STRING Genomics 11.0 database [31]. The network was visualized using the Cytoscape 3.6.0 program [32].

### Correlation and co-expression analysis of lncRNAs and mRNAs

LncRNA target gene network was used to analyze the roles of lncRNAs. Cytoscape 3.6.0 software was used to create networks of lncRNA and target genes and they were predicted in *cis* and *trans.* The Pearson correlation coefficients (PCCs) between lncRNAs and their linked mRNAs were calculated to identify co-expression analysis. Certain indicators demonstrated strong correlation, and the important role of lncRNA or mRNA in the network is determined by the degree of correlation. The absolute value of Pearson’s correlation coefficient (r2) > 0.90 and *p*-value < 0.05 were set for subsequent networking of lncRNA and mRNA [6].

### Quantitative reverse transcription PCR validation

Total RNAs were extracted from goat skin samples from the three groups and used for quantitative reverse transcription PCR (RT-qPCR). First strand cDNA was obtained using a One-step cDNA synthesis kit (Bio-Rad, USA) according to manufacturer’s instructions and was followed by subjection to quantification with GAPDH as endogenous control using standard SYBR Green PCR Kit (Bio-Rad) on the Bio-Rad CFX96 Touch™ Real Time PCR Detection System. The quantitative PCR was carried out following protocols: 95 °C for 35 s, followed by 42 cycles of 95 °C for 5 s and the optimized annealing temperature at 60 °C for 35 s and extension at 72 °C for 35 s. The primers used are listed in Table 12. Biological and technical replication was performed in triplicate for each sample. Relative gene expression was calculated using the 2^-ΔΔCt^ method and quantified relative to GAPDH. Student’s t-test were used for statistical analysis and *p*-value < 0.05 as significant. Values were expressed as means ±SD, * *P* < 0.05, ** *P* < 0.01.

## Results

### Overview of high-throughput sequencing

After extracting total RNA from three Jiangnan cashmere goats in the anagen (An), catagen (Cn), and telogen (Tn) phases, we constructed nine transcriptome libraries of cashmere goat skin samples and sequenced the RNA. Quality control findings revealed a total of 109.40Gb clean data and the percentage of Q20 of each sample was > 98.18% and the percentage of Q30 base of each sample was > 94.62%. The GC content ranged between 46.09 and 47.42% (Table 1). The numbers 1, 2 and 3 in the sample names signify the 1–3 cashmere goats. According to the quality control results, the sequencing results were reliable and adequate for further data processing. Based on the comparative results, differential splicing predictions assessment, gene structure optimization analysis, and novel gene extraction were conducted to uncover 10,054 new genes, of which 2587 were functionally annotated. 228 DEGs were discovered based on the level of gene expression in various samples and their functional annotation and enrichment analysis were performed. There were 27,740 lncRNAs discovered, with 256 DE lncRNAs.

**Table 1:** RNA-Seq quality control result

ID	READ SUM	BASE SUM	GC(%)	N(%)	Q20(%)	Q30(%)
An-1	42,348,243	12,644,980,448	47.00	0	98.42	95.21
An-2	37,058,161	11,051,625,508	47.41	0	98.54	95.58
An-3	39,272,819	11,719,721,120	46.92	0	98.35	95.03
Cn-1	44,432,825	13,244,353,818	46.85	0	98.18	94.62
Cn-2	37,226,658	11,116,926,562	46.50	0	98.51	95.43
Cn-3	36,714,406	10,961,219,362	46.10	0	98.59	95.60
Tn-1	45,820,476	13,680,058,242	46.40	0	98.48	95.37
Tn-2	42,256,910	12,603,845,766	46.82	0	98.39	95.13
Tn-3	41,468,904	12,390,529,736	46.50	0	98.51	95.41

After mapping the clean reads to goat reference genome (*Capri hircus* ARS1) (Table 2), comparison of each growth cycles proceeded. The three comparing groups were: (1) anagen vs catagen, (2) catagen vs telogen and (3) telogen vs anagen. It was demonstrated by limiting the *q*-value to < 0.05. Most of the DEGs were downregulated in the first and third group (Table 3). Functional annotations of DEGs and statistical results of the genes annotated by the gene for each differential expression is shown in Table 4.

**Table 2:** Reference genome alignment read statistics

ID	TOTAL READS	MAPPED READS	UNIQ MAPPED READS	MULTIPLE MAPPED READS	Reads Map to ‘+’	Reads Map to ‘-’
An-1	84,696,486	82,034,935(96.86%)	80,090,317(94.56%)	1,944,618(2.30%)	40,493,216(47.81%)	40,734,236(48.09%)
An-2	74,116,322	71,933,700(97.06%)	70,038,566(94.50%)	1,895,134(2.56%)	35,414,125(47.78%)	35,686,086(48.15%)
An-3	78,545,638	75,901,736(96.63%)	74,231,526(94.51%)	1,670,210(2.13%)	37,522,695(47.77%)	37,727,620(48.03%)
Cn-1	88,865,650	85,667,796(96.40%)	83,995,291(94.52%)	1,672,505(1.88%)	42,335,644(47.64%)	42,590,460(47.93%)
Cn-2	74,453,316	72,186,065(96.95%)	70,795,057(95.09%)	1,391,008(1.87%)	35,743,062(48.01%)	35,899,322(48.22%)
Cn-3	73,428,812	71,309,587(97.11%)	69,906,385(95.20%)	1,403,202(1.91%)	35,282,068(48.05%)	35,445,462(48.27%)
Tn-1	91,640,952	88,875,344(96.98%)	87,231,194(95.19%)	1,644,150(1.79%)	43,958,663(47.97%)	44,189,339(48.22%)
Tn-2	84,513,820	82,006,488(97.03%)	80,269,594(94.98%)	1,736,894(2.06%)	40,500,070(47.92%)	40,726,197(48.19%)
Tn-3	82,937,808	80,546,632(97.12%)	79,025,282(95.28%)	1,521,350(1.83%)	39,910,813(48.12%)	40,074,042(48.32%)

**Table 3:** Statistical table of the number of DEGs

#DEG Set	DEG Number	Up-regulated	Down-regulated
Cn-1,Cn-2,Cn-3 vs An-1,An-2,An-3	51	16	35
Cn-1,Cn-2,Cn-3 vs Tn-1,Tn-2,Tn-3	27	18	9
Tn-1,Tn-2,Tn-3 vs An-1,An-2,An-3	150	52	98

**Table 4:** Functional annotation for DEGs among the different stages

#DEG Set	Total	COG	GO	KEGG	KOG	NR	PFAM	SWISS-PROT	EGGNOG
Cn-1,Cn-2,Cn-3 vs An-1,An-2,An-3	41	13	37	31	22	41	39	32	38
Cn-1,Cn-2,Cn-3 vs Tn-1,Tn-2,Tn-3	24	5	18	19	11	24	19	17	19
Tn-1,Tn-2,Tn-3 vs An-1,An-2,An-3	132	30	116	103	69	128	120	90	114

**Fig. 1:**
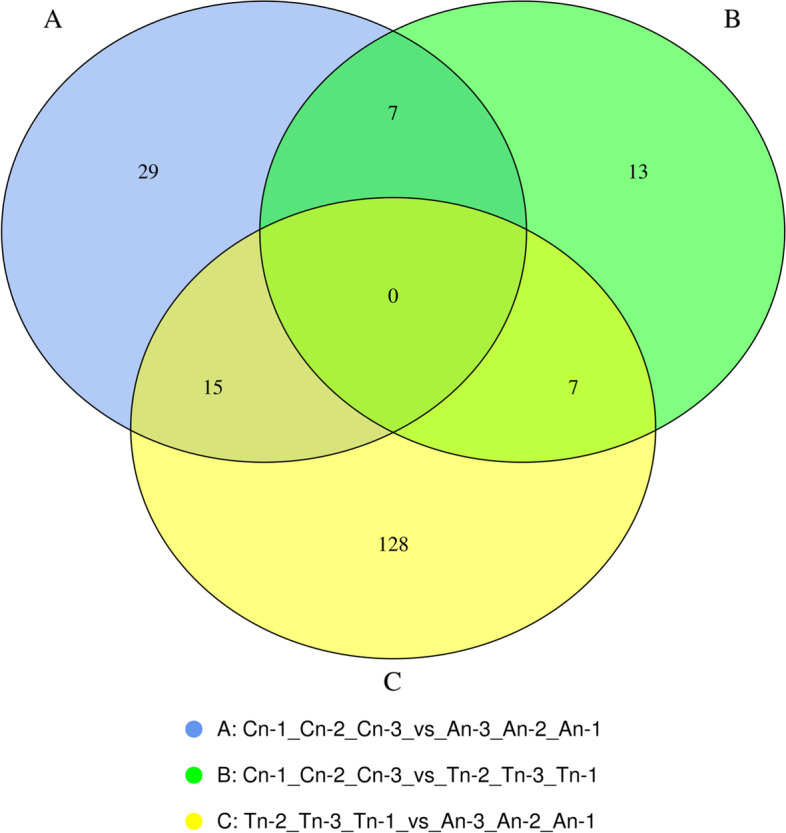
Identification of mRNAs in skin tissues of Jiangnan cashmere goat. The number of mRNAs that are unique to each comparison group, as well as the number of mRNAs that are shared by all comparison groups. Each circle indicates a unique combination of differential expression

The DEGs were depicted in a Venn diagram **(**Fig. 1**).** The number of DEGs was highest in telogen and anagen, and gene expression varied significantly in the telogen stage compared to the other phases.

**Fig. 2:**
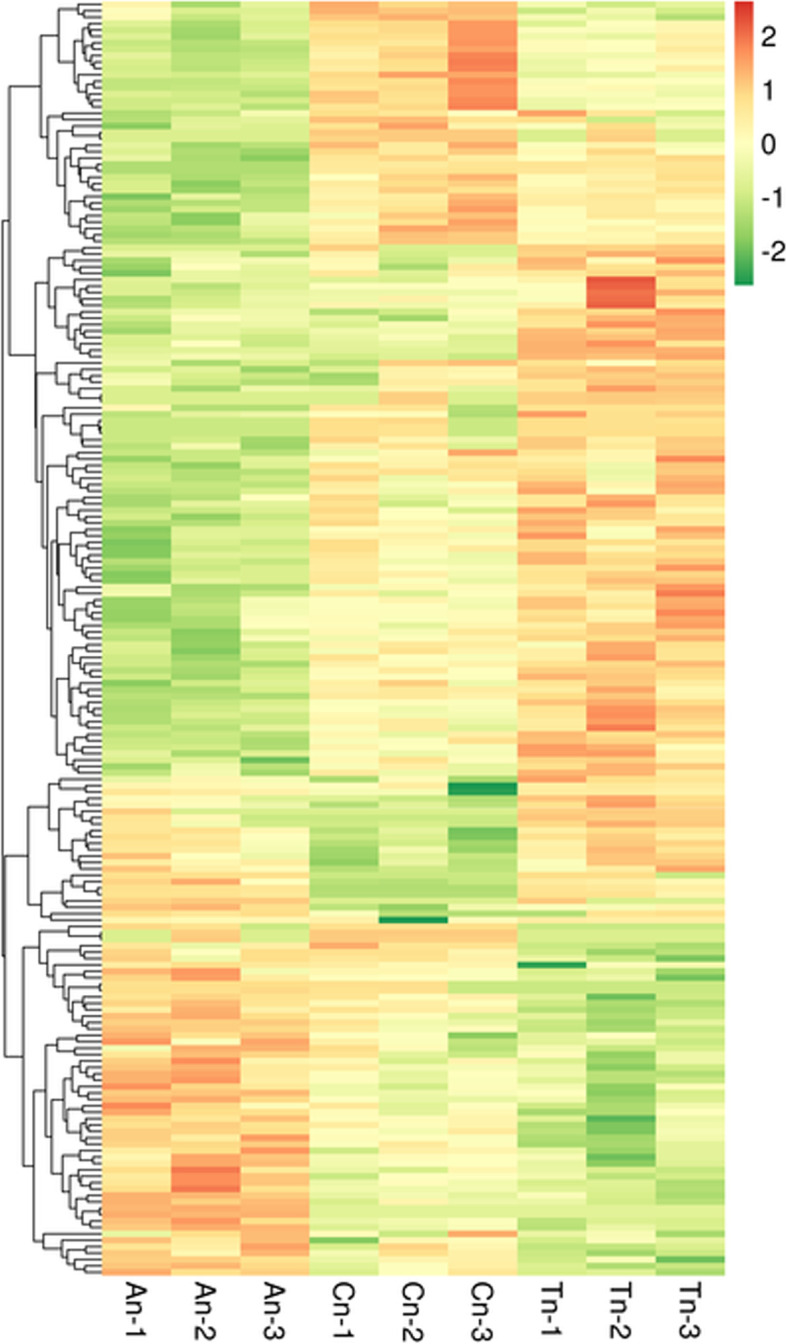
Cluster heatmap of DE mRNA on the basis of their expression values. Orange and green indicate high and low expressions, respectively. The colors denote differential expression levels (log_2_ (fold change) ≥1 and *p*-value< 0.05)

The mRNAs were grouped using hierarchical clustering based on their similarities in gene expression patterns across the three groups (Fig. 2). Gene expression pattern in the telogen phase appeared to be significantly different compared to the other phases.

**Fig. 3:**
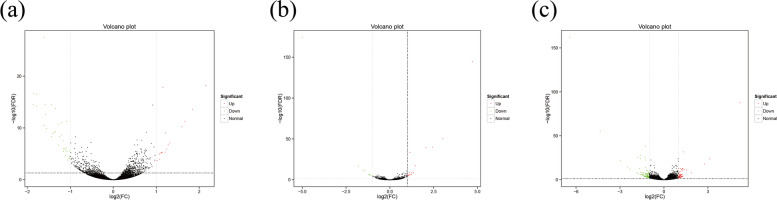
Volcano plots of DE mRNAs at different cycles of SHF development. **a** catagen vs anagen (**b**) catagen vs telogen (**c**) telogen vs anagen. The y-axis indicates the −log10(FPKM+ 1) values, the green points in the figure represent the down-regulated, the red dot represents the up-regulated, and the black represents the non-difference expression

Volcano maps were plotted to analyze the differences in gene expression levels between groups as well as statistical significance of the differences Fig. 3.

### Enrichment of DEGs

**Fig. 4:**
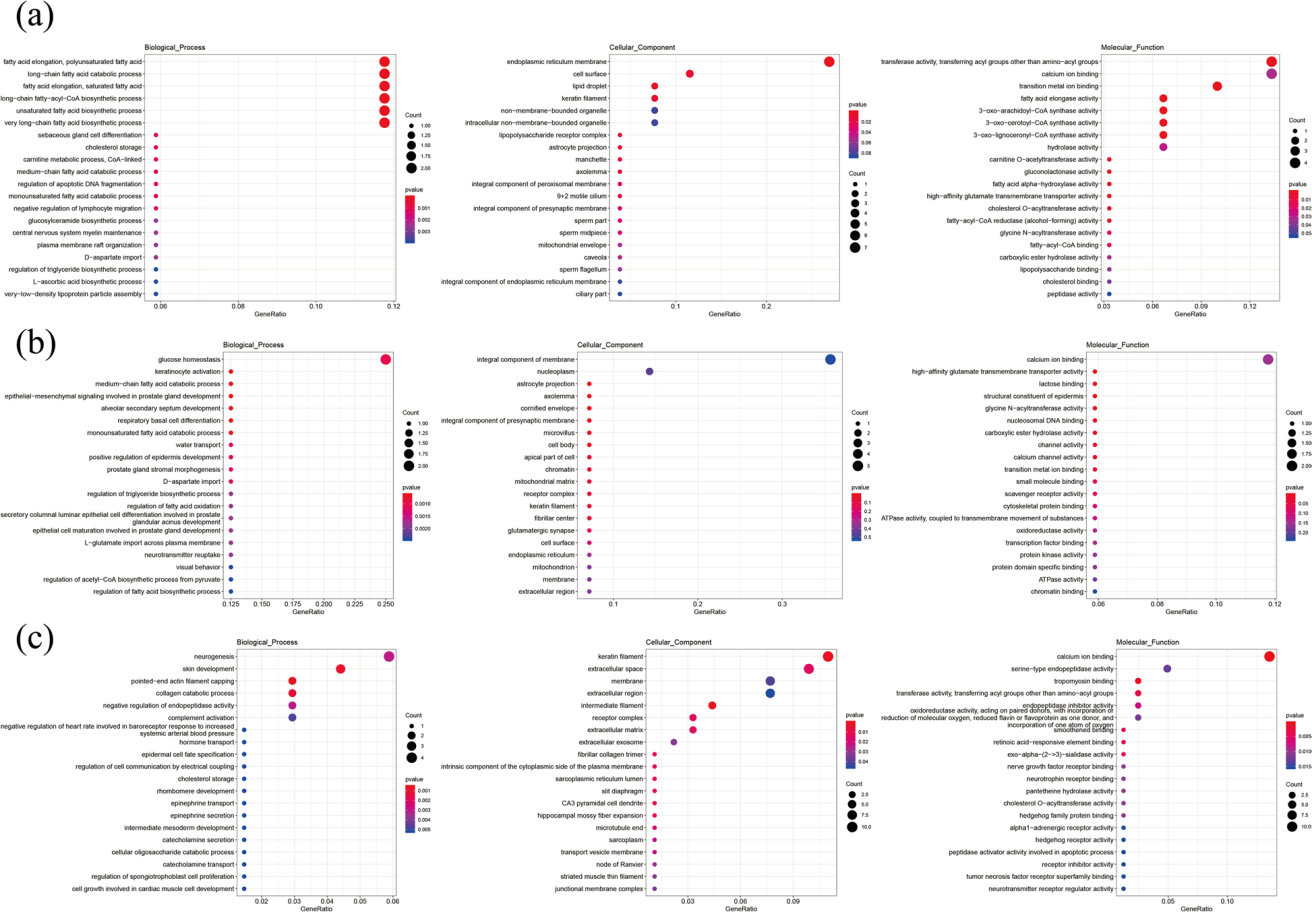
Enriched GO terms for DE mRNAs (*p*-value < 0.01). **a** catagen vs anagen (**b**) catagen vs telogen (**c**) telogen vs anagen

Gene ontology (GO) and Kyoto Encyclopedia of Genes and Genome (KEGG) analyses were performed to better understand the biological processes and pathways involved in cashmere HF development. Following the comparison of DEGs across groups, the top enriched GO terms (*p*-value < 0.05) were analyzed. Among the groups the DEGs were related to biological processes like fatty acid elongation, polyunsaturated fatty acid (GO:0034626), long-chain fatty acid catabolic process (GO:0042758), fatty acid elongation, saturated fatty acid (GO:0019367), long-chain fatty-acyl-CoA biosynthetic process (GO:0035338) and unsaturated fatty acid biosynthetic process (GO:0006636), keratinocyte activation (GO:0032980), medium-chain fatty acid catabolic process (GO:0051793), skin development (GO:0043588), pointed-end actin filament capping (GO:0051694), collagen catabolic process (GO:0030574) and molecular function terms like calcium ion binding (GO:0005509), transition metal ion binding (GO:0046914), fatty acid elongase activity (GO:0009922), high affinity glutamate transmembrane transport activity (GO:0005314) as well as the cellular function terms like lipid droplet (GO:0005811), keratin filament (GO:0045095), suggesting their developmental and maintenance functions in cashmere HF cycle. The top 20 GO terms among the various cycles are shown in (Fig. 4).

**Fig. 5:**
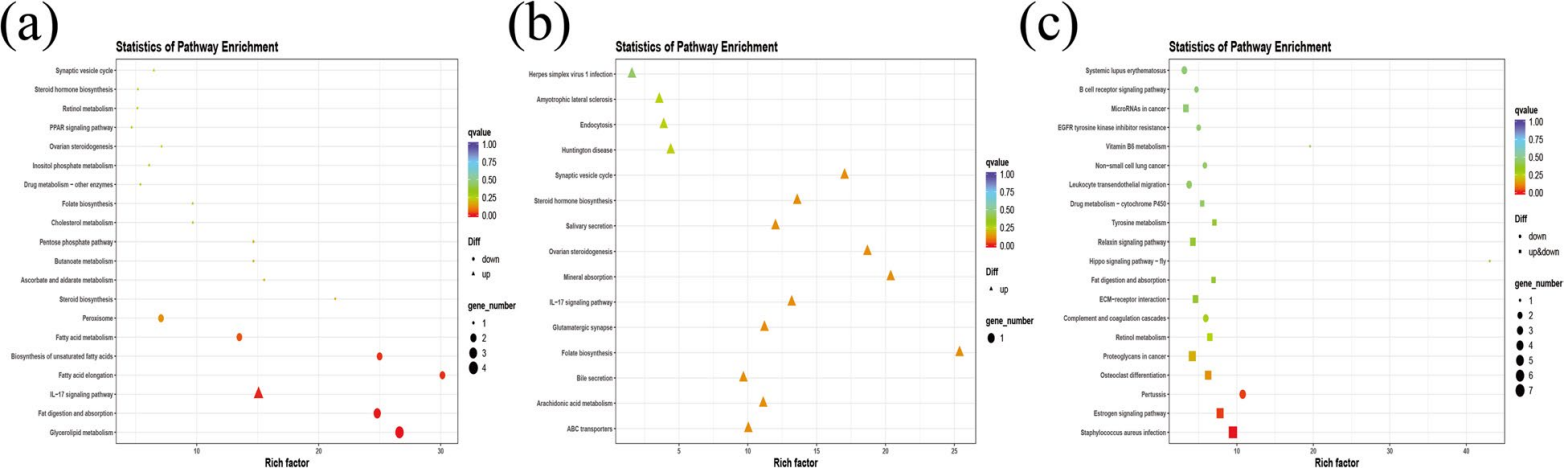
Enriched KEGG pathway for DE mRNAs (*p*-value < 0.05). **a** catagen vs anagen (**b**) catagen vs telogen (**c**) telogen vs anagen. The ratio of mRNAs enriched in the pathway among those identified in the pathway is indicated by the rich factor

According to the KEGG pathway, there were significant changes in the physiological processes in the various phases of the SHF development and cycling. Catagen vs Anagen showed enrichment of 35 KEGG pathways, including Arachidonic acid metabolism, glycolipid metabolism, fatty acid elongation, fatty acid metabolism, folate biosynthesis and Hippo signaling pathway. Catagen vs telogen showed enrichment of 15 KEGG pathways, including folate biosynthesis, mineral absorption, IL-17 signaling pathway, glutamatergic synapse and arachidonic acid metabolism. Telogen vs anagen showed enrichment of 143 KEGG pathways, including Hippo signaling pathway, WNT signaling pathway, JAK-STAT signaling pathway, NF-kappa B signaling pathway, Th17 cell differentiation, Th1 and Th2 cell differentiation, PI3K-Akt signaling pathway, VEGF signaling pathway, Arachidonic acid metabolism and IL-17 signaling pathway. The top 20 KEGG pathways [33–35] among the different cycles were shown in (Fig. 5).

### Identification of lncRNAs

**Fig. 6:**
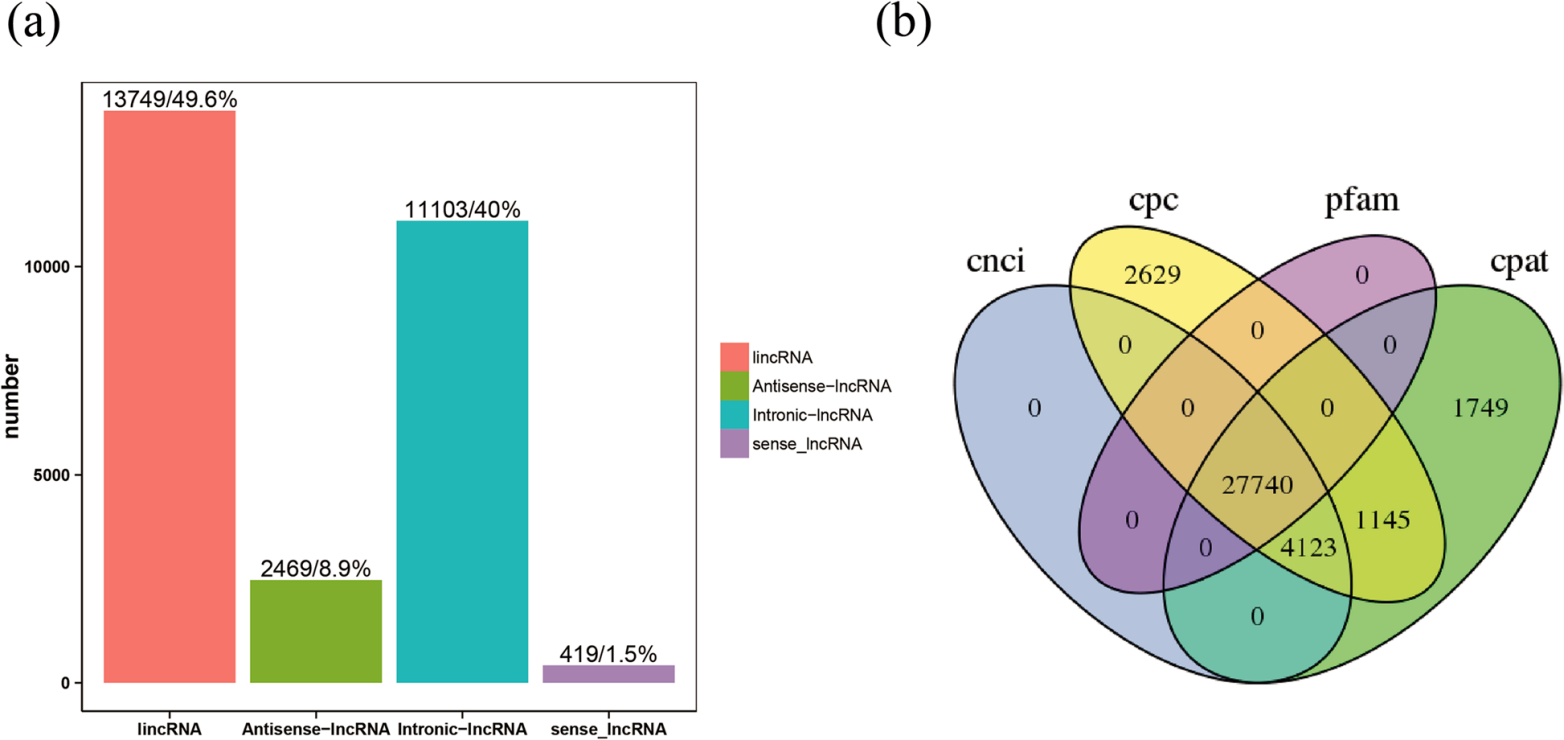
Identification of long noncoding RNAs (lncRNAs) in Jiangnan cashmere goat skin tissues. **a** Bar chart showing the four categories of lncRNA. **b** Venn diagram showing the number of lncRNAs with coding potential analysis by CNCI, CPC, CPAT and PFAM

37,386 lncRNAs were discovered and their genomic contexts were examined based on their similarities to local protein-coding genes. They were classified into (13749) long intergenic RNAs (lincRNAs), (2469) Antisense-lncRNA, (11103) Intronic-lncRNA, (419) sense lncRNA (Fig. 6a). After excluding candidate lncRNAs with coding potential using the software CNCI, CPC, CPAT and PFAM-scan protein domain analysis, 27,740 lncRNAs were identified (Fig. 6b).

The number of lncRNA expressed by the differences in each group is demonstrated in Table 5. Tn-1,Tn-2,Tn-3 vs An-1,An-2,An-3 had the highest DEGs.

**Table 5:** Differentially expressed lncRNA

#DEG Set	DEG Number	Up-regulated	Down-regulated
Cn-1,Cn-2,Cn-3 vs An-1,An-2,An-3	61	22	39
Cn-1,Cn-2,Cn-3 vs Tn-1,Tn-2,Tn-3	65	36	29
Tn-1,Tn-2,Tn-3 vs An-1,An-2,An-3	163	66	97

**Fig. 7:**
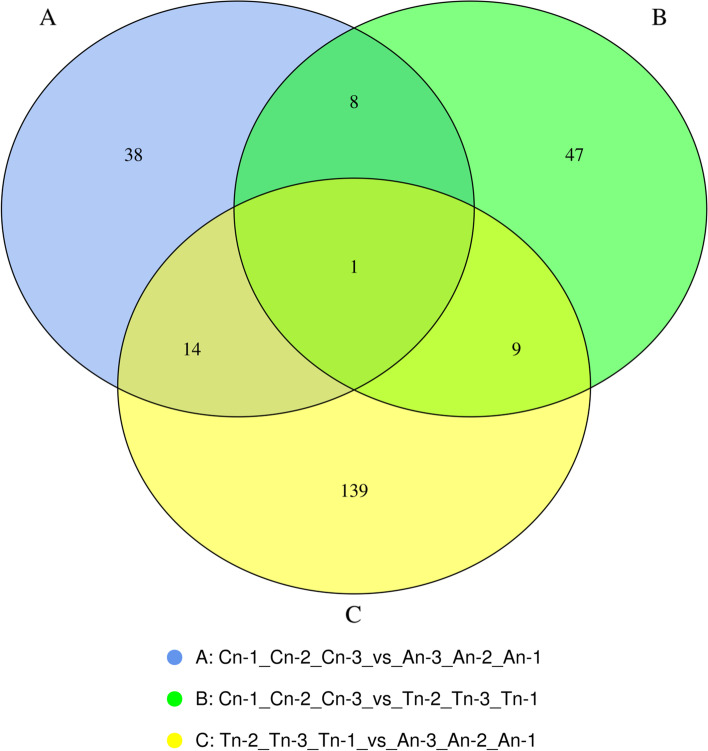
Venn diagram showing the number of DE lncRNA with differences and similarities among the comparison groups. **A** catagen vs anagen **B** catagen vs telogen **C** telogen vs anagen

The number of lncRNAs with unique and common differences between the comparison groups were plotted. Each set of DE lncRNA was demonstrated in Venn diagram (Fig. 7).

**Fig. 8:**
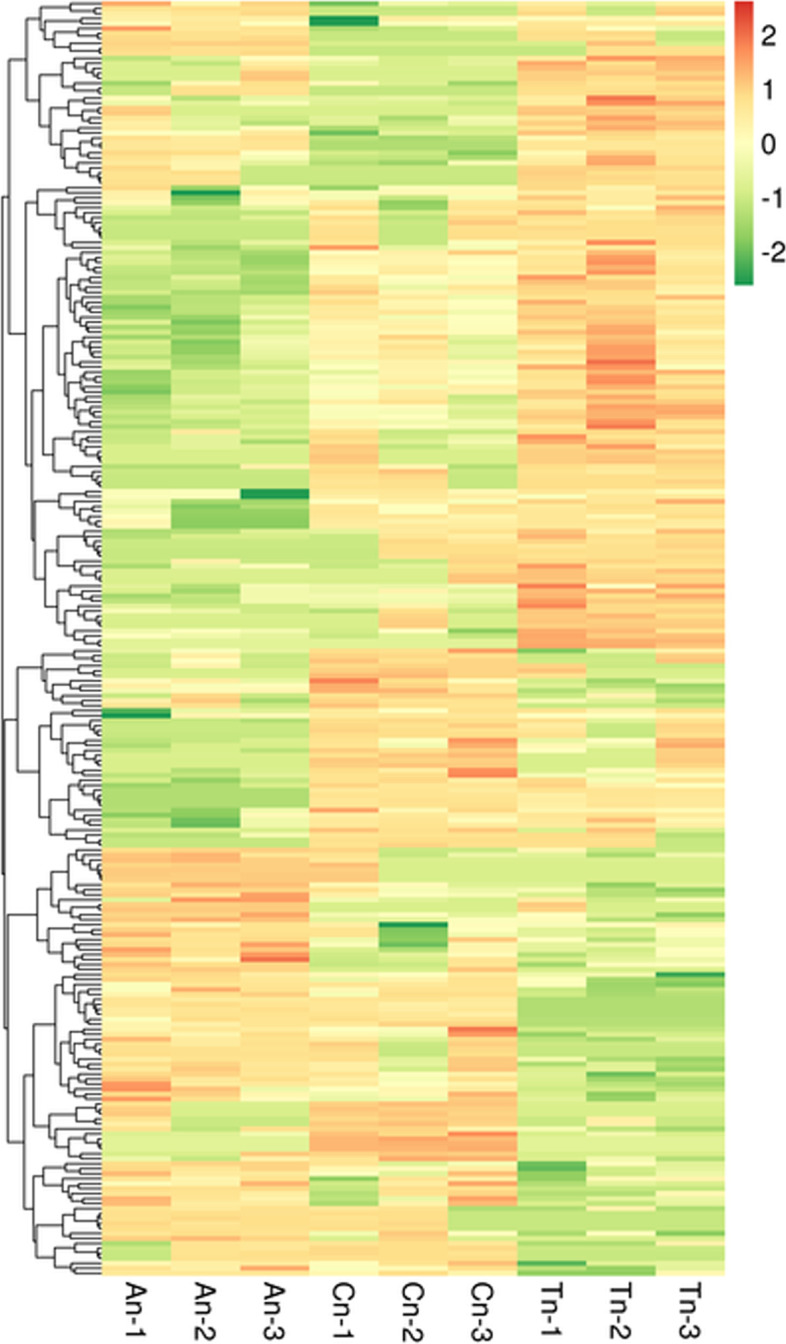
Clustered heat map of DE lncRNAs on the basis of their expression values. Orange and green indicate high and low expressions, respectively. The colors denote differential expression levels (log_2_ (fold change) ≥1 and *p*-value< 0.05)

The DE lncRNAs were grouped using hierarchical clustering based on their similarities in gene expression patterns across the three groups (Fig. 8).

### DE lncRNA functional classification analysis in Jiangnan cashmere goats

**Fig. 9:**
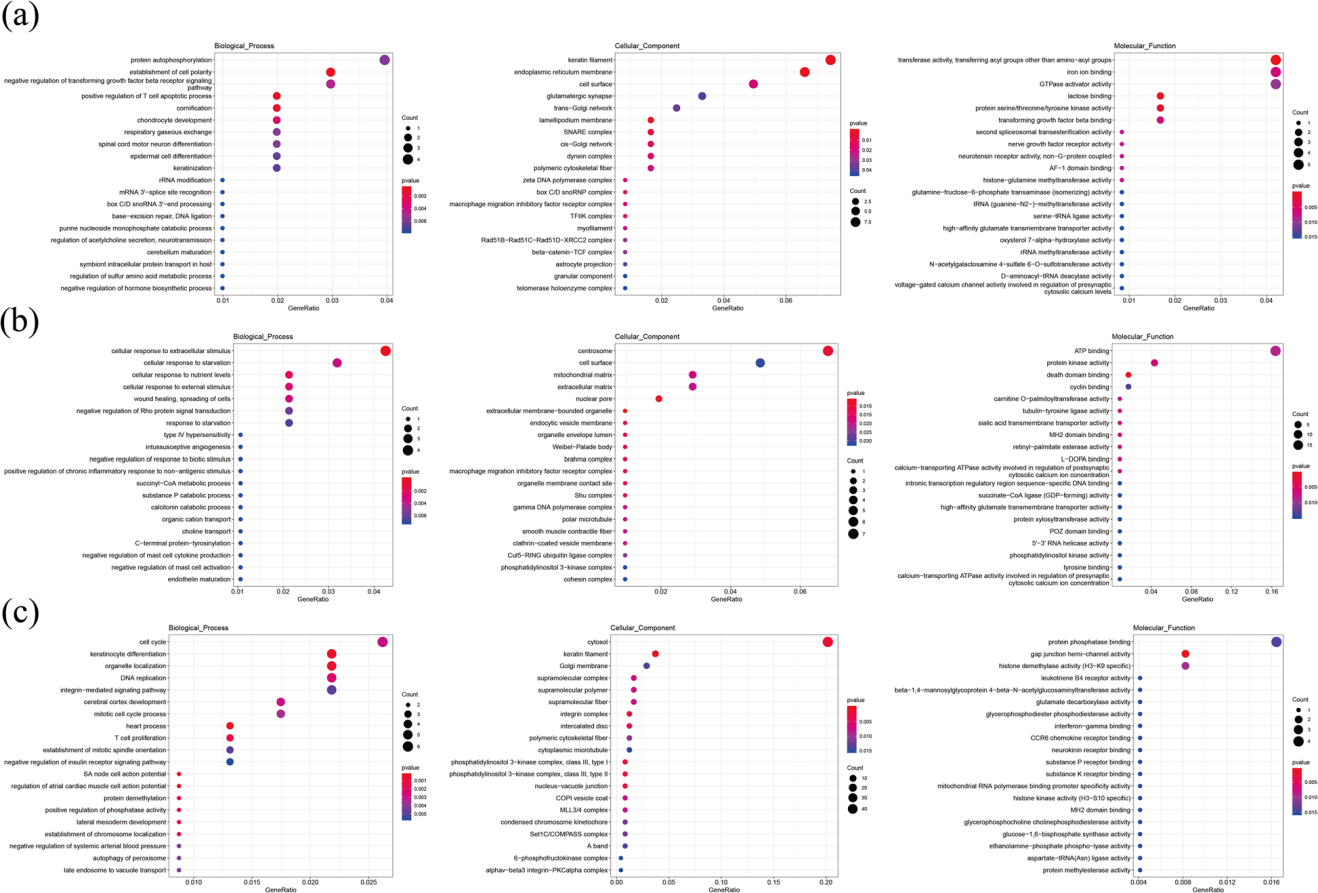
Enriched GO terms for lncRNAs *cis*-target genes (*p*-value < 0.01) (**a**) catagen vs anagen (**b**) catagen vs telogen (**c**) telogen vs anagen

EdgeR, GO and KEGG analyses were used to investigate the functional clustering characteristics of DE lncRNAs between cashmere goat seasons. The lncRNAs *cis* target genes were enriched in important GO terms related to HF development. Among the groups the lncRNAs *cis* target genes were related to biological processes such as cornification (GO:0070268), epidermal cell differentiation (GO:0009913), keratinization (GO:0031424), lipid biosynthesis process (GO:0008610), keratinocyte differentiation (GO:0030216), regulation of BMP signaling pathway (GO:0030510), lipid modification (GO:0032102), epidermal growth factor receptor signaling pathway (GO:0007173), positive regulation of ERK1/2 cascade, Wnt signaling pathway (GO:0016055), integrin-mediated signaling pathway, Notch signaling pathway (GO:0007219), positive regulation of vascular endothelial growth factor production (GO:0010575) and hippo signaling (GO:0035329). The cellular component terms showed enrichment in keratin filament (GO:0045095), endoplasmic reticulum membrane (GO:0005789), integrin complex (GO:0008305), growth cone (GO:0030426) and actin filament (GO:0005884). The molecular functions among the various cycles showed enrichment in transforming growth factor beta binding (GO:0050431), growth factor receptor binding (GO:0070851), NF-kappaB binding (GO:0051059), collagen binding (GO:0005518), beta-catenin binding (GO:0008013), actin filament binding (GO:0051015) and microtubule binding; indicating their potential developmental functions and maintenance activities in cashmere cycling. The top 20 GO terms among the various cycles are shown in (Fig. 9).

**Fig. 10:**
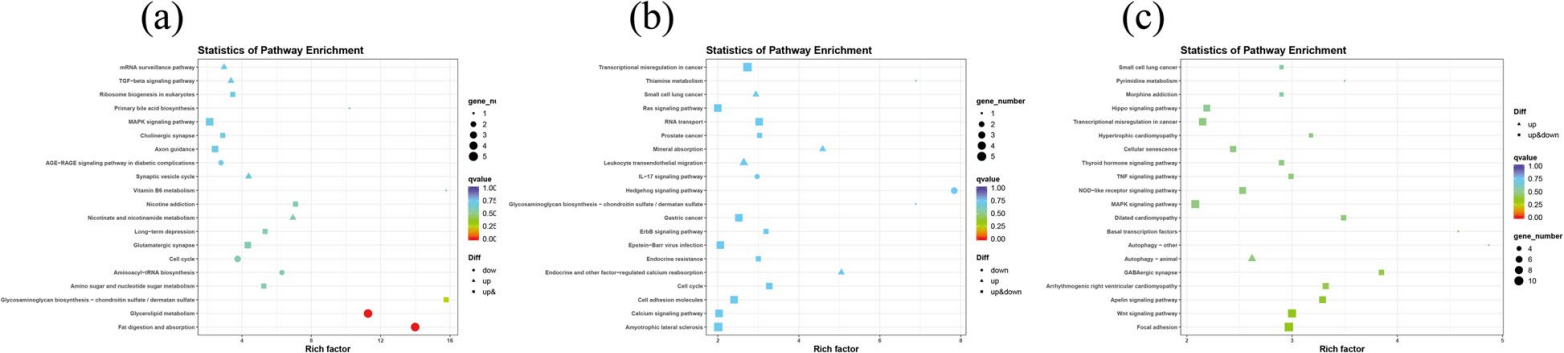
Top 20 significantly *(p*-value < 0.05) enriched KEGG pathway for lncRNAs *cis*-target genes **(a)** between catagen and anagen **(b)** between catagen and telogen **(c)** between telogen and anagen. Rich factor represents the ratio of DE lncRNA *cis-*target genes enriched in the pathway among genes annotated in the pathway

According to the KEGG pathway, there were significant changes in the physiological processes occurring at the various phases of cashmere goat hair follicle development and cycling. Some of the observed KEGG pathways belonged to conventional techniques linked with the cashmere HF cycle. Catagen vs Anagen showed enrichment of 108 KEGG pathways including pl3k-Akt signaling pathway, Hippo signaling pathway, IL-17 signaling pathway, fatty acid metabolism, Notch signaling pathway, TGF-beta signaling pathway, glycerolipid metabolism, fat digestion and absorption, MAPK signaling pathway, glutamatergic synapse and cell cycle. Catagen vs telogen showed enrichment of 171 KEGG pathways including MAPK signaling pathway, Th1 and Th2 cell differentiation, PPAR signaling pathway, Notch signaling pathway, NF-Kappa B signaling pathway, PI3K-Akt signaling pathway, Wnt signaling pathway, IL-17 signaling pathway and Hedgehog signaling pathway. Telogen vs anagen showed enrichment of 222 KEGG pathways including JAK-STAT signaling pathway, Th17 cell differentiation, Hedgehog signaling pathway, Arachidonic acid metabolism, NF-Kappa B signaling pathway, Notch signalling pathway, pl3k-Akt signaling pathway, IL-17 signaling pathway, Hippo signaling pathway and Wnt signaling pathway. The top 20 KEGG pathways [33–35] with the highest representation of lncRNAs *cis*-target genes are shown in (Fig. 10).

**Fig. 11:**
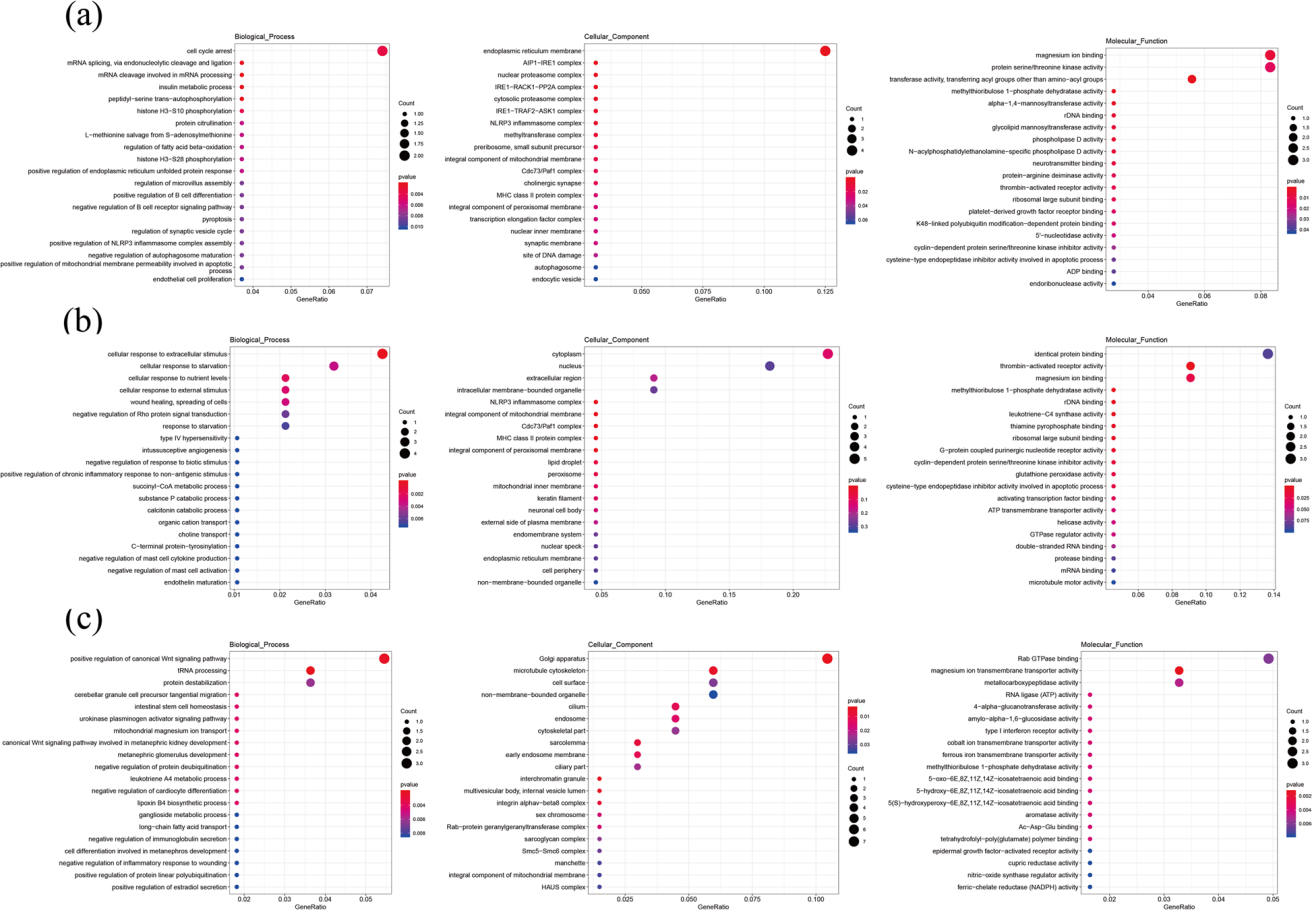
Enriched GO terms for lncRNAs *trans-*target genes (*p*-value < 0.01) (a) catagen and anagen (b) catagen and telogen (c) telogen and anagen

The lncRNAs trans target genes were enriched in important GO terms relating to HF development. Among the groups the lncRNAs trans target genes are related to biological processes such as positive regulation of NF-kappaB transcription factor activity, cell cycle arrest (GO:0007050), positive regulation of B cell differentiation (GO:0045579), fatty acid beta-oxidation (GO:0006635), regulation of ERK1/2 cascade (GO:0070372), lipid transport (GO:0006869), positive regulation of NF-KappaB transcription factor activity (GO:0051092), lipid transport (GO:0006869), fatty acid transport (GO:0015908), positive regulation of MAPK cascade (GO:0043410), positive regulation of canonical Wnt signaling pathway, germ cell development (GO:0007281) and integrin mediated signaling pathway (GO:0007229). The cellular component terms showed enrichment in autophagosome (GO:0005776), keratin filament (GO:0045095), integrin alphav-beta8 complex (GO:0034686), actin cytoskeleton (GO:0015629), cilium (GO:0005929), microtubule cytoskeleton (GO:0015630) and sarcolemma (GO:0042383). The molecular functions among the various cycles showed enrichment in magnesium ion binding (GO:0000287), protein serine/threonine kinase activity (GO:0004674), transcription factor activity, sequence-specific DNA binding (GO:0003700), transferase activity, transferring acyl groups other than amino-acyl groups (GO:0016747), ATP binding (GO:0005524), calcium ion binding (GO:0005509), magnesium ion transmembrane transporter activity (GO:0015095), zinc ion binding (GO:0008270), metal ion binding (GO:0046872) and protein binding (GO:00055115). The top 20 GO terms among the various cycles are shown in (Fig. 11).

**Fig. 12:**
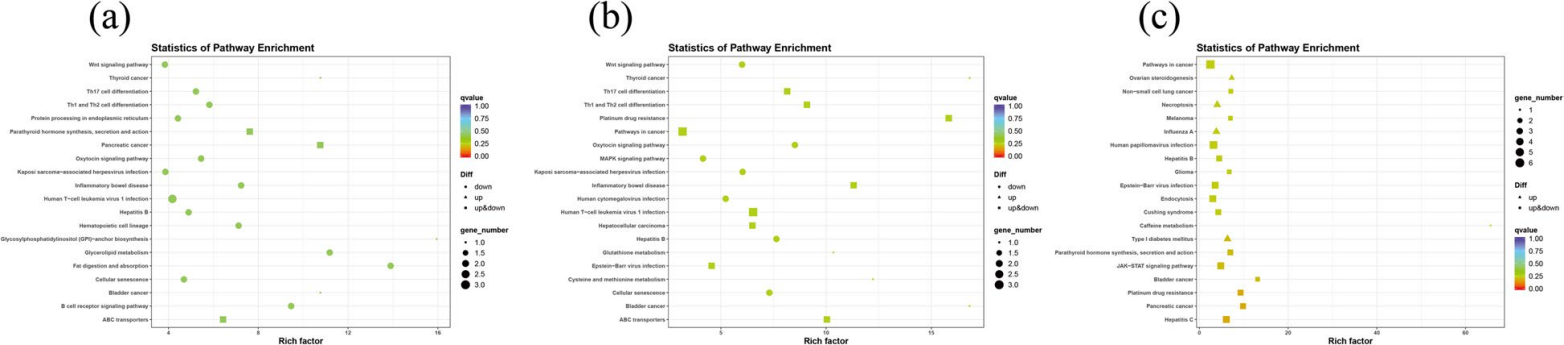
Top 20 significantly *(p*-value < 0.05) enriched KEGG pathway for lncRNAs *trans*-target genes (**a**) between catagen and anagen (**b**) between catagen and telogen (**c**) between telogen and anagen. Rich factor represents the ratio of DE lncRNA *trans*-target genes enriched in the pathway among genes annotated in the pathway

According to the KEGG pathways, there were significant changes in the physiological processes occurring at various phases of cashmere goat hair follicle development and cycling. Some of the observed KEGG pathways belonged to conventional pathways associated with cashmere hair follicle cycle. Catagen vs Anagen showed enrichment of 118 KEGG pathways including Pl3K-Akt signaling pathway, MAPK signaling pathway, WNT signaling pathway, Th17 cell differentiation, Th1 and Th2 cell differentiation and glycerolipid metabolism. Catagen vs telogen showed enrichment of 93 KEGG pathways including Pl3K-Akt signaling pathway, Th17 cell differentiation, Th1 and Th2 cell differentiation, WNT signaling pathway, MAPK signaling pathway and Glutathione metabolism. Telogen vs anagen showed enrichment of 139 KEGG pathways including WNT signaling pathway, MAPK signaling pathway, NF-kappaB signaling pathway, Th17 cell differentiation, Th1 and Th2 cell differentiation, cell cycle, Arachidonic acid metabolism, Glutamatergic synapse, Pl3K-Akt signaling pathway and calcium signaling pathway. The top 20 KEGG pathways [33–35] with the highest representation of lncRNAs *trans*-target genes are shown in (Fig. 12).

DE lncRNA screening was carried out and the top ten upregulated and downregulated genes, as well as their potential target genes were chosen in Cn1, Cn2, Cn3 vs An3, An2, An1 (Table 6), Cn1, Cn2, Cn3 vs Tn1, Tn2, Tn3 (Table 7) and Tn1,Tn2,Tn3 vs An1,An2,An3 (Table 8). The DE lncRNA’s targets were predicted using both *cis* and *trans* principles. The Fold Change ≥2 and FDR < 0.05 served as screening criteria. The fold change multiple represents the ratio of expression between two samples (groups). We found that some lncRNAs, *MSTRG.46401.1, MSTRG.4712.2, MSTRG.48192.1* predominantly have *trans* target genes in the KRT family and is directly associated to the growth of goat hair follicles, and KRT dysregulation could result in hair diseases Table 6. *MSTRG.48150.1*, *MSTRG.48153.1, MSTRG.48154, MSTRG.48155* have *cis* target genes mainly *WNT8B,* which is a member of WNT gene family, involved in regulating hair follicles morphogenesis.

**Table 6:** DE lncRNA of Cn1, Cn2, Cn3 vs An3, An2, An1

LncRNA Transcript	FDR	Log2FC	regulated	Targeted genes name
MSTRG.5652.5	1.90006103665003e-17	4.04	up	LOC102177175, BMP8A, HEYL, PABPC4, HPCAL4, NT5C1A
MSTRG.11609.2	6.40188743057225e-24	4.54	up	CHST11
MSTRG.31376.2	8.81625887122821e-16	3.87	up	PPFIBP2, OLFML1
MSTRG.28834.1	1.21921928769418e-30	4.98	up	ATP9A, SALL4
MSTRG.5451.2	1.8776452147065e-06	2.72	up	SH3BP4
MSTRG.43744.5	0.00111889108767857	2.15	up	IRF4, DUSP22
MSTRG.41648.1	0.00217814842838439	2.08	up	FBLN5, DTD2, ABCB10, DHX33, LOC102183805, NIF3L1, NUBPL, PLD1, LOC102172428, PIGM, STPG1, SLC25A43, NIPSNAP1, GPR33, HEATR5A
MSTRG.17024.7	0.00118619751262157	2.15	up	CACNA1A, LYL1, NFIX, STX10, TRMT1, IER2, NACC1,
MSTRG.30926.1	1.9944247107694e-06	2.07	up	SLC1A2, CD44
MSTRG.44016.6	2.74529933999282e-06	2.68	up	LOC102172428
MSTRG.36440.2	2.5077155037872e-16	−4.02	down	FBL, LGALS15, LEUTX, FCGBP, DYRK1B, LOC102184749
MSTRG.9934.2	9.43074830882305e-27	−4.71	down	AGMO
MSTRG.23575.2	5.18823089644824e-131	−8.41	down	HSPB3, CD27, MAP 4 K4
MSTRG.16556.4	3.56881345173044e-11	−3.44	down	CHSY3, CDKN1A,
MSTRG.14493.1	1.57436912596199e-28	−4.94	down	ARHGAP24
MSTRG.26725.15	7.13742462085733e-08	2.95	down	KCNRG, TRIM13, LOC108637304, SPRYD7
MSTRG.40547.1	2.08921303003978e-10	−3.22	down	TRIO, LOC102187998, LOC102178740
MSTRG.20801.17	5.67094750934937e-15	−3.77	down	DYNLT1, SYTL3, TMEM181, TULP4
MSTRG.53049.2	7.42540015765072e-15	−3.75	down	COL4A5
MSTRG.52737.5	4.93503638255332e-07	−2.82	down	GRIA3

**Table 7:** DE lncRNA of Cn1, Cn2, Cn3 vs Tn1, Tn2, Tn3

LncRNA Transcript	FDR	Log2FC	regulated	Targeted genes name
MSTRG.23343.1	2.32701721027499e-53	6.01	up	MCC
MSTRG.52443.14	1.13508635802771e-15	3.85	up	PNPLA4, CDKN1A
MSTRG.31376.2	5.690517204483e-14	3.66	up	PPFIBP2, OLFML1, LOC102186611
MSTRG.42958.2	8.61759786500729e-32	5.22	up	HSPB3, SUCLG2, LOC108636657, LOC108636665, LOC108636663, SUCLG2
MSTRG.43056.2	1.05666921943582e-15	3.95	up	PTPRG, LOC102181343
MSTRG.45465.3	4.18671313242219e-11	3.40	up	GAREM1
MSTRG.20755.3	2.72144441727715e-51	5.96	up	ARID1B
MSTRG.53213.2	2.25129625736325e-27	4.70	up	DIAPH2
MSTRG.18792.2	1.1199048792559e-45	5.72	up	AGTPBP1
MSTRG.25400.5	9.1487884974327e-10	3.20	up	C111H9ORF50
MSTRG.23575.2	1.05256376149683e-143	−8.66	down	HSPB3, CD27, MAP 4 K4
MSTRG.19273.7	2.19351796254616e-19	−4.23	down	ZNF618
MSTRG.19729.2	4.61501069502626e-13	−3.58	down	–
MSTRG.8535.2	1.06119699270715e-12	−3.56	down	SLC13A4, FAM180A, NUP205, C4H7orf73
MSTRG.4846.1	1.63667928143256e-62	−6.38	down	SP9, SCRN3, GPR155, CIR1, OLA1
MSTRG.3468.2	6.07510628131043e-07	−2.77	down	FAM124B
MSTRG.42157.2	3.48521496294758e-06	−2.64	down	TECPR2, MOK, TECPR2, CINP, WDR21, ZNF839
MSTRG.840.3	8.30524395727213e-08	−2.92	down	GSK3B, LOC108638286, NR1I2
MSTRG.4445.2	1.45675256051493e-07	−2.87	down	LYPPD6B
MSTRG.50051.1	3.40978840175212e-07	−2.82	down	TBCE, B3GALNT2, GNG4

**Table 8:** DE lncRNA of Tn1,Tn2,Tn3 vs An1,An2,An3

LncRNA Transcript	FDR	Log2FC	regulated	Targeted genes name
MSTRG.38847.1	0.000709948926488846	2.35	up	MYL4, CDC27, KANSL1, ITGB3
MSTRG.3468.2	4.41823500140194e-12	3.84	up	FAM124B, CUL3,
MSTRG.42043.2	6.50059750861016e-21	4.66	up	VRK1
MSTRG.7303.2	0.0181354825490137	1.88	up	BCL9, LOC108638397, ACP6, GJA5
MSTRG.8652.7	0.0138939980061938	1.92	up	COPG2, SLURP1, MEST, TSGA13
MSTRG.41332.1	0.000596147828281096	1.85	up	ANKRD34C, RASGRF1, TMED3
MSTRG.34360.13	0.0257527628791044	1.80	up	CUX2
MSTRG.12955.1	0.0198689230997933	1.84	up	
MSTRG.32111.1	0.000158760794190582	1.85	up	BIRC3, YAP1
MSTRG.17024.7	2.39262610333967e-05	2.72	up	CACNA1A, LYL1, NFIX, STX10, TRMT1, IER2, NACC1
MSTRG.48847.2	1.22634131003528e-27	−5.04	down	CDKN1A
MSTRG.23997.2	6.7092482263365e-15	−4.06	down	SRBD1
MSTRG.30305.2	4.02723848180614e-09	−3.42	down	KCNK9
MSTRG.35616.4	8.25692069159788e-10	−3.46	down	BANP
MSTRG.21003.1	2.43033150987906e-28	−5.15	down	AFDN
MSTRG.4480.3	1.1274696442633e-08	−3.35	down	CACNB4, NEB, ARL5A
MSTRG.25400.3	3.37913242429768e-15	−4.20	down	C11H9orf50, LOC102172072
MSTRG.43056.2	1.15965618888042e-15	−4.31	down	LOC102181343, PTPRG
MSTRG.34860.6	1.19856618469239e-20	−4.81	down	MAML3, ANKRD39
MSTRG.14493.1	2.56009768999488e-12	−3.84	down	ARHGAP24

### Characteristic analysis of lncRNA and mRNA

**Fig. 13:**
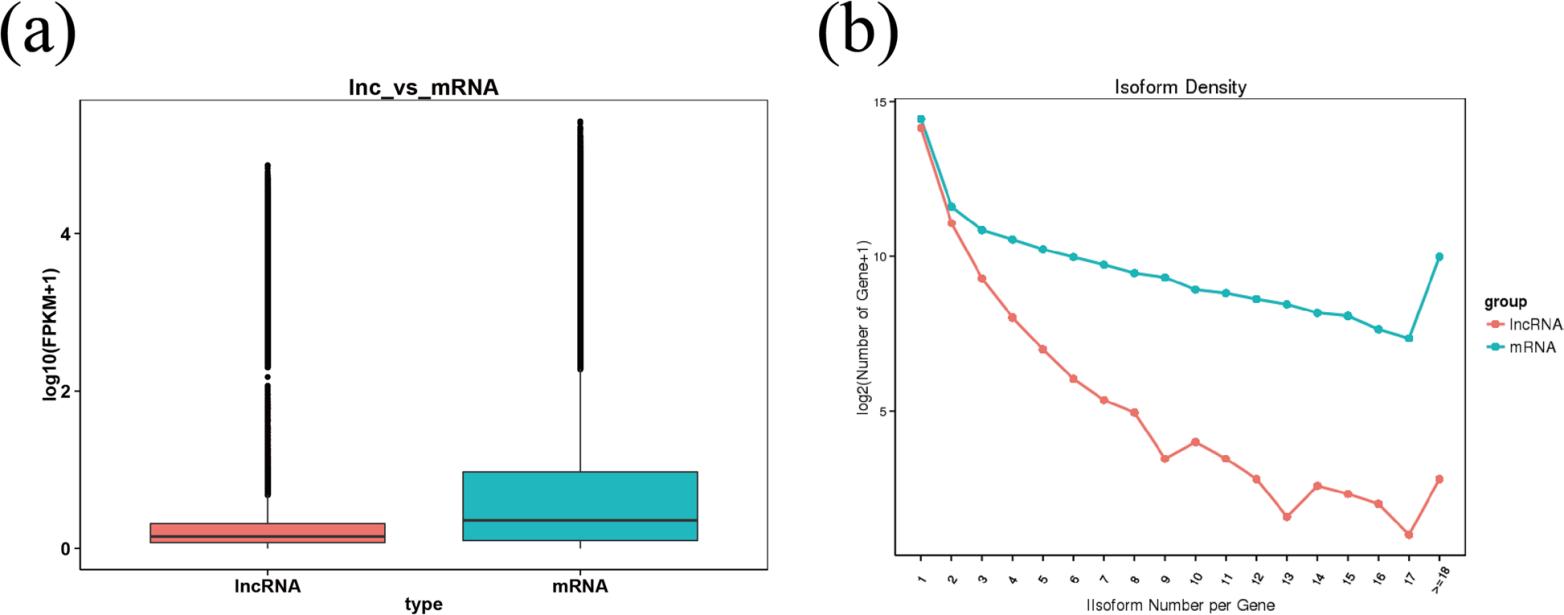
Comparison of genomic primary differences and expression levels between lncRNAs and mRNAs in cashmere goat hair follicle. **a** The expression levels were indicated by log10(FPKM+ 1) in the lncRNAs and mRNAs. **b** Comparison of variable shear isomers for lncRNAs and mRNAs

Transcription length, exon number, ORF length, expression level, variable shear isomer, and interactive analysis were used to analyze the differences and characteristics of lncRNA and mRNA in terms of understanding the structural features of genes in cashmere goats (Fig. 13). The length distribution study revealed that lncRNAs were longer than mRNAs. The prediction of *cis* and *trans* target genes for DE lncRNAs were used to provide better understanding to the functions of the lncRNAs at various stages of development.

The mRNAs compared with lncRNAs expression levels and mRNAs and lncRNAs variable shear isomer comparative analysis are shown in Fig. 13.

**Fig. 14:**
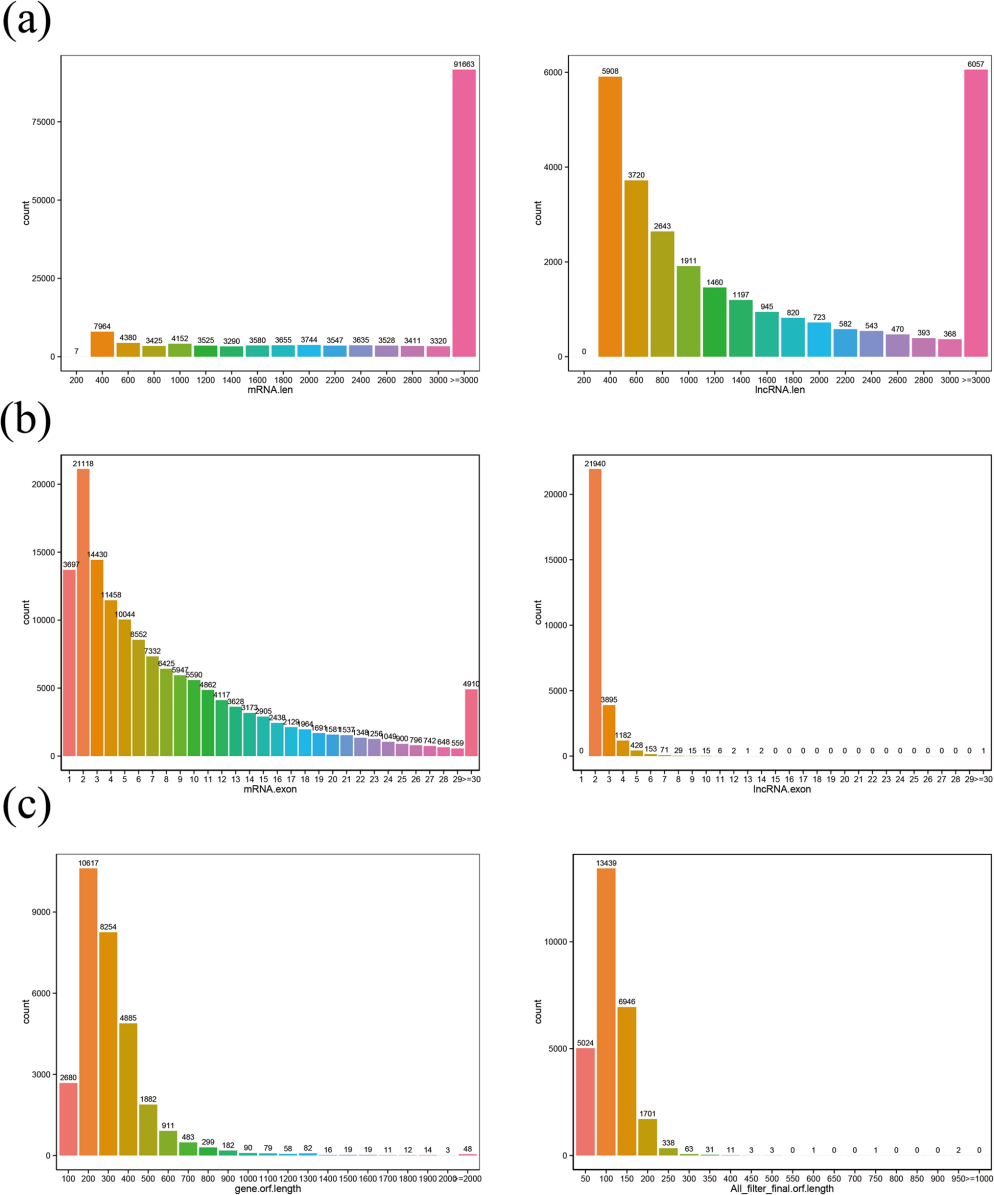
Comparison of genomic architecture and expression level between lncRNAs and mRNAs in cashmere goat hair follicle. **a** Distribution of transcript length in lncRNAs and mRNAs in goat skin. The horizontal axis indicates the length of transcripts and the vertical axis represents density (**b**) Distribution of the number of exons in the mRNAs and lncRNAs. **c** Distribution of the number of open reading frames (ORFs) in the mRNAs and lncRNAs. The ORFs were identified using Estcan

To understand the differences in architecture and expression levelsbetween the mRNAs and lncRNAs, different analyses were done. The mRNAs compared with lncRNAs length, lncRNAs exons and ORF length of lncRNAs Fig. 14.

### DE lncRNA and DE mRNA interactive analysis

The expression of lncRNAs and mRNAs in different interactive analysis, in relation to the similarities and differences between the RNAs in different cycles.

**Fig. 15:**
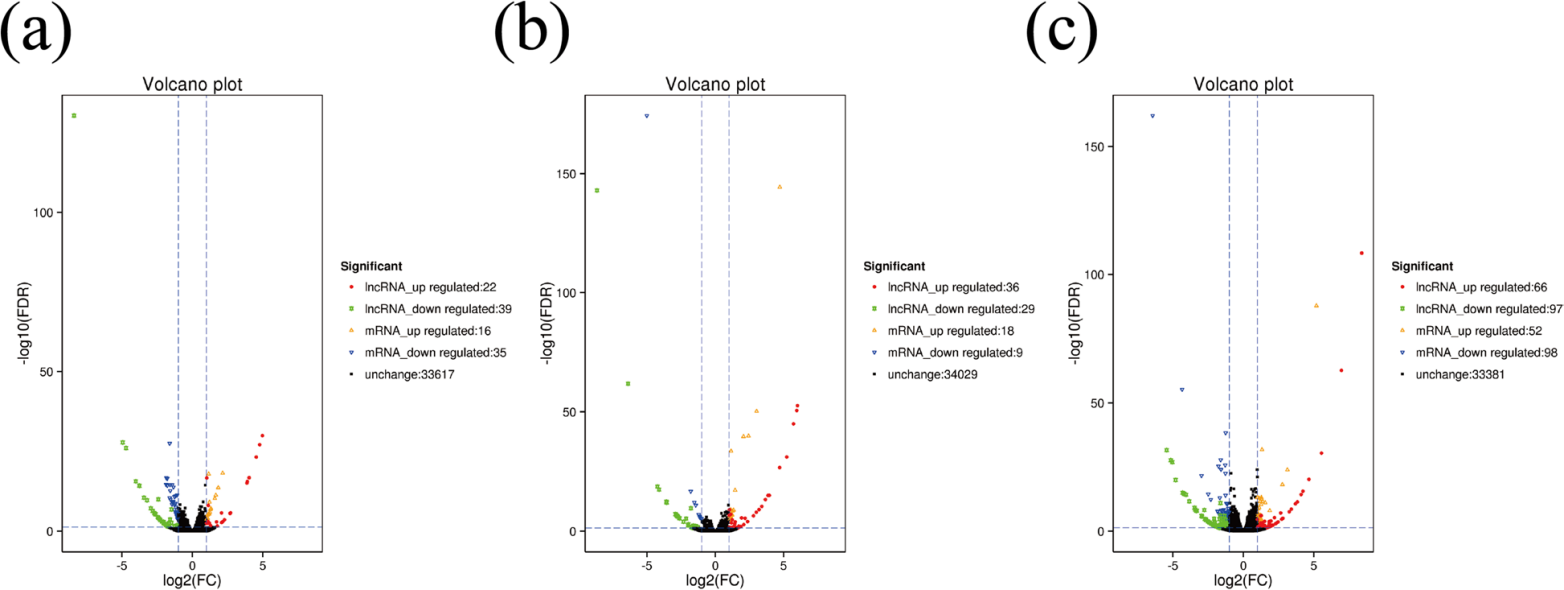
Volcano plot of differentially expressed (DE) lncRNA and mRNA (log2(fold change) ≥1 and *p*-value < 0.05). **A** Catagen vs Anagen (**B**) Catagen vs Telogen (**C**) Telogen vs Anagen. The y-axis indicates the −log10(FPKM+ 1) values. Red represent upregulated lncRNA, green represents downregulated lncRNA, orange represents upregulated gene, blue represents downregulated gene, and black represents non-differential expression gene

Volcano maps were plotted to analyze the relationships in gene expression levels between groups as well as statistical significance of those relationships. The relationships between the groups were expressed in the volcano maps in Fig. 15.

**Fig. 16:**
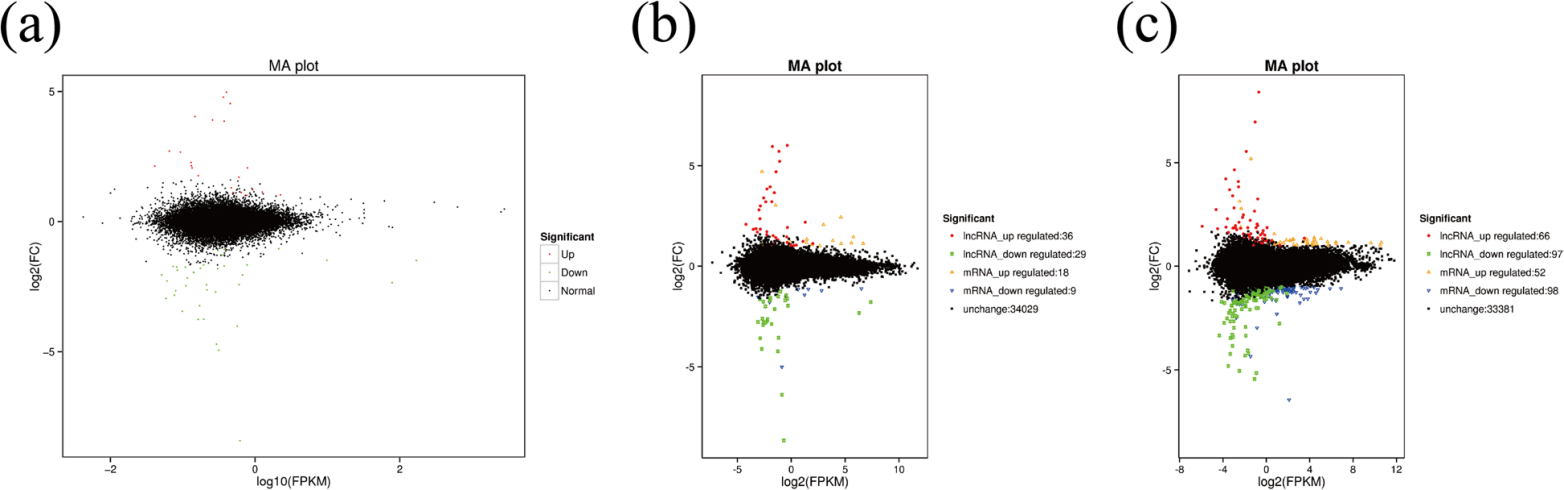
MA interactive diagram of differences in the expression of lncRNA and mRNA (**A**) Catagen vs Anagen (**B**) Catagen vs Telogen (**C**) Telogen vs Anagen. The x-axis indicates log2 (FPKM). Red represents upregulated lncRNA, green represents downregulated lncRNA, orange represents upregulated genes, blue represents downregulated genes, and black points represent genes that express no significant difference

MA interactive diagrams were plotted to analyze the relationships in gene expression levels between groups. The relationships between the groups are expressed in the MA diagram in Fig. 16**.**

**Fig. 17:**
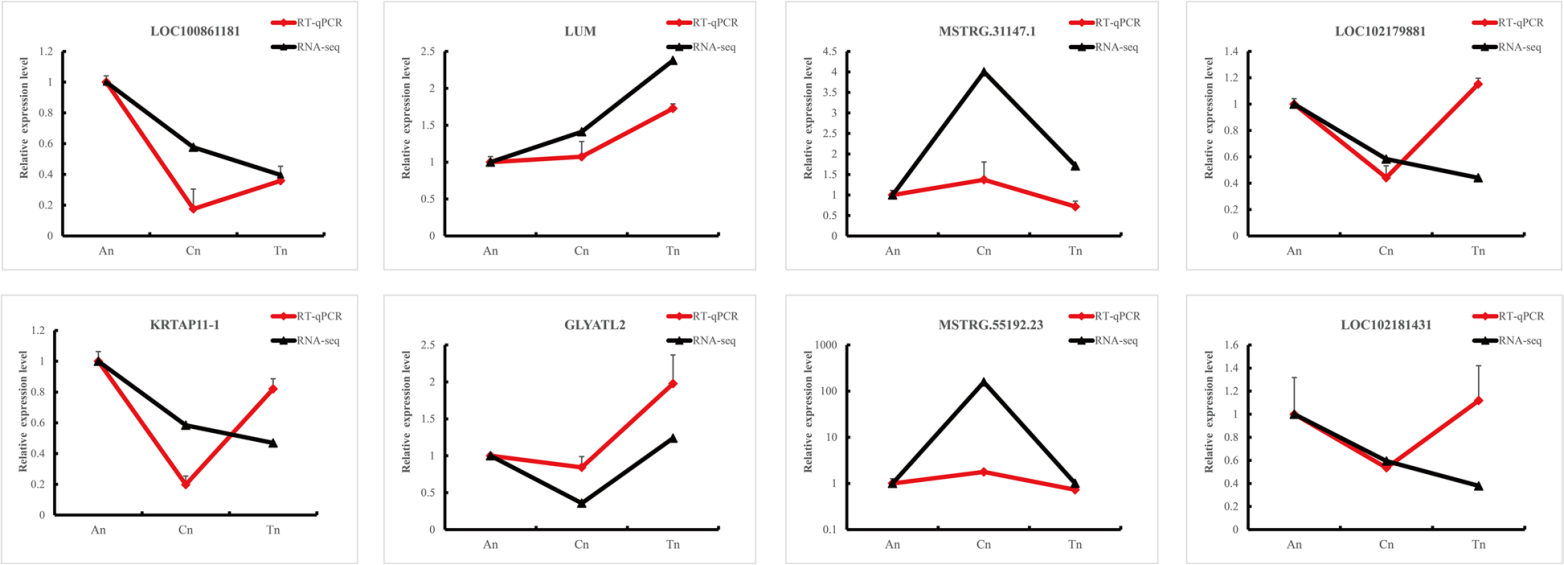
QPCR validation of RNA-Seq. Eight mRNAs and lncRNAs were selected for validation including *LOC1021179881, LOC102181431, LOC100861181, MSTRG.55192.23, MSTRG.31147.1, KRTAP11–1, LUM* and *GLYATL2.* The x-axis indicates the three main cycles of SHF and the y-axis indicates lncRNA and mRNA relative expression level (mean ± SEM)

The sequencing results were validated using RT-qPCR. Figure 17 depicts the end results. It was normal that the expression of *LOC1021179881* and *LOC102181431* between RNA-Seq and qPCR were inconsistent. Some genes/ lncRNAs were low-abundant while sensitivity of RNA-Seq and qPCR were different which caused the difference in expression.

## Discussion

Cashmere goats exist as a type of cyclical molting animals used to study photoperiod-dependent hair follicle morphological changes [9]. Their SHFs account for the important part of the skin that produce fine variable cashmere fleece in repetitive cycles, traversing the phases of growth (anagen), regression induced by apoptosis (catagen) and relative quiescence (telogen) [3, 9, 24]. The recurrent cycles are regulated by extensive and well-orchestrated interactions between follicular epithelial cells and mesenchymal cells of dermal papilla (DP) to necessitate the dynamic synthesis of various stimulatory and inhibitory signals [36, 37]. WNT, Notch, BMP/TGF, NF-kappa B, HGF, Shh, and EDA/EDAR signaling pathways are among those implicated in critical signaling pathways which intricate interplay and interdependence of HF development and cycling [32, 38–41]. At least, three key developmental signaling pathways (WNT, SHH, and NF-kB/EDAR) are essential for hair follicle growth and maintenance [3, 17]. LncRNAs do not encode protein [19, 20]. They are at least 200 bp long and include conserved secondary structures [38]. Numerous lncRNAs have been shown to have critical roles in biological processes including apoptosis, proliferation and differentiation [38, 42]. They interact with DNAs, RNAs, and proteins to regulate gene expression through different interconnected mechanisms including cell cycle regulation, splicing regulation, gene imprinting, mRNA degradation and stabilization, and translational regulation [22, 38, 43, 44]. The general characteristics of lncRNAs and mRNAs reveal that, they are relatively comparable in terms of being 5^′^ capped, spliced and polyadenylated and lncRNAs are much less evolutionarily conserved, contain fewer exons and are less abundantly expressed [38]. The specific function of lncRNAs and mRNAs in HF development and cycling have not been extensively analyzed and as a result remain poorly understood. Since RNA expression levels change overtime and across tissues, transcriptome sequencing is regarded ideal for exploring gene expression variations [45]. In this study, we used RNA-Seq technology to investigate the SHF development cycle in cashmere goats. We analyzed lncRNAs and mRNAs in SHF development among the three main cycles (anagen, catagen and telogen). We identified 256 DE lncRNAs, some of which were *trans* and *cis* target genes and 228 DE mRNA involved in SHF development and cycling. The main limitation of the study is that, goat genomic annotation is incomplete.

The randomly selected lncRNAs and mRNAs used for validating the RNA-Seq results revealed the following findings **(**Table 9**).** *GLYATL2* is involved in fat synthesis [46] and linked to hair follicle morphogenesis and cycling as it is involved in lipid modification of signaling proteins such as Hedgehogs (HHs) and WNTs [47]. *Lum* regulate development, growth and healing through the functional activities of cytokines. *Lum* together with *Lgals1* and *Apoa1* collectively enhance the adult fibroblast competence for HF neogenesis [48]. *Lum/ Lgals1/ Apoa1* may help promote Wnt signaling [48]. *Lum* serves as a key node in the regulatory network and is required for collagen fibrillogenesis in skin homeostasis [10]. *LOC102181431* (*KRTAP13–1*) is part of PMG protein family which is characterized by anagen specific KAPs and play important role in phosphorylation which regulates the formation of keratin filament cell cycle [49]. *KRT* and *KRTAP* constituent the major structural proteins of the hair fiber and sheath and their products serve as essential commodity for fleece quality and cashmere goat hair morphology [5]. *KRTAP* gene expression modulate the fineness and density of cashmere as well as other certain features [50]. *LOC102179881* (*KRT33A*) is primarily expressed in hair follicle and highly expressed in its cortex, where it interacts with keratinization and developmental biology pathways [51]. *KRTAP11–1* genetic variation may regulate its expression, protein structure and posttranslational modifications and subsequently affect wool fiber structure and wool traits [52]. *MSTRG.31147.1* has target gene *PADI1* is involved in hair follicle differentiation and may take part in transcriptional regulation for hair follicle keratin synthesis [53, 54]. *MSTRG.55192.23* targets *COL1A1* is involved in processes and pathways that promote cyclic growth of HF [32].

**Table 9:** Primers used in RT-qPCR analysis

ID	No	GENE NAME	SEQUENCE	PRODUCT LENGTH
MSTRG.31147.1	L3	MSTRG.31147.1	F:TTGTCCTGCTCAGTGAGTGAC R:ATGGGTCCTCTTCATGTCTGC	107
MSTRG.55192.23	L4	MSTRG.55192.23	F:GTACCCCAAGGCTCCCTCT R:GCTTCCCTGTACCGCAGAC	154
gene36	M1	LOC100861181	F:TGCCACGATGACTCGTTTCT R:CCATAACCAAGGGGAGAGCC	135
gene35	M2	KRTAP11–1	F:TCCACCATCTGCCAACCTAC R:GCTGGCAGGTCATGGAATCT	125
gene19597	M3	LOC102179881	F:TGTCTCACGTACTCGGTGTG R:GAAGGCTGGTCCCAGAAGTG	117
gene47	M4	LOC102181431	F:TTCAGACGCCTGGGTTACGG R:CAACTCCTGGAGGCAAAGAAGGT	92
gene4866	M11	LUM	F:GAAAGCAGGGTCGAGACAGTA R:ACAGTTTGGTGATGACCTCCC	166
gene14546	M12	GLYATL2	F:AGGCATCGAGTGGTGACAAG R:CCCAACTCCCAGTGCTCATT	142
GAPDH	M13	GAPDH	F:GGCGTGAACCACGAGAAGTAT R:ATGGCGTGGACAGTGGTCAT	143

Functional annotation of DEGs were analyzed using KEGG and GO database. DEGs significantly enriched in GO analysis included *HSD3B5, GTF2IRD1, GDAP10, GYS3, GSN, CDK2AP1, CD151, ADGRE5* etc. (Fig. 4). KEGG pathway analysis (Fig. 5) showed that *HOXA4, LTα, COQ7, ASSP12, KCND2, FSHR, SF3B4, GTL11* were enriched in Hippo (ko04391), VEGF (ko04370), Pl3k-Akt (ko04151), NF-kappa B (ko04064), Wnt (ko04310) and Hedgehog (ko04340) signaling pathway [33–35]. These findings imply that the mRNAs may play key roles in the signaling networks across distinct hair follicle developmental stages of cashmere goats. *COQ7* has been shown to regulate a number of developmental and physiological mechanisms such as apoptosis, cell cycle, gene expression, rhythmic behavior and aging [55]. It was found in NF-kappa B (ko04064) signaling pathway, exclusively in telogen and anagen phase and may function as a key developmental signaling pathways. *HOXA4* and *LTα* were related to Hippo (ko04391)/ (ko04392) signaling pathway, respectively. *LTα* may activate NF-kappaB-signaling pathway and implicate the control of normal hair follicle development and the pathophysiology of certain hair disorders [56, 57].

In the Cn1, Cn2, Cn3 vs An3, An2, An1 phase, a variety of DEGs in hair follicle development were identified **(**Table 10**).** The results showed that epidermal differentiation complex (EDC) genes were upregulated. EDCs promote keratinocyte differentiation singly and/or combination, leading to epidermal and hair follicle differentiation. They include S100 family; *LOC102191570, LOC108633164, S100A7A* and function as innate immune regulatory elements [58]. *S100A7A* is mainly found in cytoplasm and nucleus of different types of cells involved in continuous regulation of cellular migration, invasion, differentiation and cell cycle progression [59]. The expression levels of *S100A7A* in normal skin of humans are usually low and in altered conditions, their expression increase [58]. The strong upregulation of *S100A7A* could be characterized by increased immune regulatory functions as a result of psoriasis and other hyperproliferative skin conditions, featuring impaired epidermal differentiation. The main regulatory pathways of *S100A7A* expression are NFkB/p38MAPK, caspase-1, and interleukin (IL)-1a [59]. *GLYATL2* is related to fat synthesis and it was significantly upregulated. *KRT4* upregulation suggests significant increase in cell proliferation to promote SHF development and also increase cashmere yield [60]. Elevated expression of *SLC1A2* suggests that it may promote hair growth and keratinocyte proliferation [61]. Dysregulation of *LOC102190037/LOC102179090/LOC102173866* may impair the essential role of lipid metabolism in goat skin functions, probably because lack of essential fatty acids could reduce water repulsion and increase trans-epidermal water loss [62]. They may exert their functions through signaling proteins such a Hedgehogs (HHS) and WNTS, which are essential for hair follicle development and cycling [47]. Downregulation of *FA2H* may disrupt synthesis of 2-hydroxylated glucosylceramide and wax diesters in sebaceous gland and cause delayed hair fiber from follicles, hyperproliferation and cyclic alopecia [63]. In Cn1, Cn2, Cn3 vs Tn1, Tn2, Tn3, *AQP8* and *KRT2* were highly functional and could play key roles in hair follicle development **(**Table 11**).** *AQP8* play significant roles in transportation of water, urea, ammonia, hydrogen peroxide and gland secretion in hair follicle development [64]. Keratins are well-known for their important involvement in hair follicle cell proliferation and differentiation [49, 65] and are predominantly found in hair cortex [66]. *KRT2* mainly participates in epidermis keratinization and its expression was lower during telogen and this causes inactive hair growth [24]. In Tn1,Tn2,Tn3 vs An1,An2,An3 a number of mRNAs played key roles in hair follicle development **(**Table 12**).** *KRT39* was upregulated and have shown to be potentially significant for regulating hair follicle fiber growth and fineness [6]. Decreased expression of β-defensins have been associated with human atopic dermatitis. *LOC102188015 (DEFB103A)* was upregulated and may play significant roles in innate immunity against bacterial skin infections [67]. *FAM167A* is transcribed in many cell lines and tissues and is suggested to exert functions in immune pathways and cell motility. It may be involved in autoimmune pathogenesis of lupus which scar hair follicles, causing hair loss [68]. *EGFL6* is mostly expressed from the epidermis and it has shown to be essential for proper alignment, touch response and av. integrin-enrichment of lanceolate complexes [69]. The downregulation of *EGFL6* may affect stable hair follicle-lanceolate complex interface responsible for tactile sensation [70]. *FAT4* has been demonstrated in human cancer, including melanoma. It performs significant function in suppressing tumor growth through activation of Hippo signaling and its genetic perturbations in hair follicle has been implicated in their development and cycling [71]. In this study, downregulation of *FAT4* may induce cell cycle arrest [72].

**Table 10:** The top 10 upregulated and downregulated DEGs of Cn1, Cn2, Cn3 vs An3, An2, An1

#ID	FDR	Log2FC	regulated
S100A7A	5.33E-12	1.67	up
LOC102191570	1.75E-07	1.28	up
LOC102189201	1.23E-06	1.24	up
GLYATL2	8.43E-10	1.22	up
KRT4	4.38E-06	1.21	up
LOC108636554	1.33E-18	1.16	up
LOC102187932	4.96E-06	1.14	up
LOC108633164	5.03E-06	1.13	up
SLC1A2	0.000105	1.08	up
FAR2	6.72E-10	−1.37	down
LOC102190037	1.03E-09	−1.40	down
SOAT1	3.26E-15	−1.43	down
LOC102179090	2.19E-13	−1.57	down
LOC102173866	3.18E-15	−1.58	down
PLIN4	3.24E-28	−1.61	down
FA2H	3.05E-17	−1.78	down
TRIL	5.51E-15	−1.78	down
AADACL3	2.18E-17	−1.85	down
THRSP	2.70E-15	−1.86	down

**Table 11:** The top 10 upregulated and downregulated DEGs of Cn1, Cn2, Cn3 vs Tn1, Tn2, Tn3

#ID	FDR	Log2FC	regulated
GLYATL2	7.89E-18	1.45	up
LOC102187932	9.89E-07	1.12	up
LOC108633164	1.61E-09	1.33	up
SLC1A2	1.83E-07	1.24	up
LOC102186944	1.34E-40	2.42	up
LOC102182197	1.08E-09	1.13	up
AQP8	2.43E-07	1.16	up
LOC102175358	2.25E-40	2.06	up
TRPV6	2.83E-34	1.15	up
PDK4	4.31E-09	1.26	up
LGALS15	1.25E-06	−1.15	down
OPCML	2.44E-06	−1.00	down
KRT2	2.44–06	− 1.12	down
CD5L	5.08E-07	−1.15	down
AADACL3	2.18E-17	−1.42	down
THRSP	2.70E-15	−1.21	down

**Table 12:** The top 10 upregulated and downregulated DEGs of Tn1,Tn2,Tn3 vs An1,An2,An3

#ID	FDR	Log2FC	regulated
KRT4	4.38E-06	1.21	up
NPTX2	4.16E-11	1.25	up
CA4	8.69E-07	1.24	up
LOC108636562	7.83E-14	1.28	up
LOC102183480	0.000101	1.24	up
KRT39	1.79E-32	1.32	up
LOC102188015	3.20E-12	1.30	up
LOC108635405	4.87E-05	3.13	up
STUM	3.93E-13	1.29	up
FAM167A	2.77E-13	1.27	up
LOC102175358	5.05E-26	−1.77	down
PDK4	7.82E-09	−1.58	down
EGFL6	2.45E-06	−1.44	down
LRP1B	8.68E-07	−1.51	down
LOC102191700	6.47E-07	−1.51	down
FAT4	4.13E-09	−1.39	down
LOC102171556	9.31E-25	−1.58	down
LOC108633257	1.06E-13	−1.36	down

LncRNA is known to play important roles in regulating development of cashmere goats and therefore serve as potential candidate genes for studying the molecular mechanisms of hair follicle morphogenesis. Despite the fact that they do not encode proteins, LncRNAs have the ability to regulate gene expression and stability of target genes [42]. The regulatory pattern characteristics of lncRNAs indicate that they may be classified as *cis* and *trans.* The *cis* regulate adjacent genes whereas *trans* regulation occur throughout the cell [20]. *MSTRG.11609.2* target gene *CHST11* has demonstrated to be positively regulated by transforming growth factor-beta (TGFβ) signaling pathway [73] and plays key roles in HF development. This study suggests that *CHST11* promotes proliferation of dermal papillae for hair growth partly through activation of WNT/β-catenin signaling of HF cells. *MSTRG.5451.2* target gene *SH3BP4* is involved in indirect regulation of cell growth, proliferation and autophagy. It negatively regulates GTPase complex and amino acid dependent mTORC1 signaling [74]. The upregulation of mTORC1 signaling by the target gene also affect WNT/ β-catenin signaling and may cause loss of HF maintenance and impaired hair growth. Although, overactive WNT signaling leads to HF degeneration in anagen initiation [75]. *MSTRG.41648.1* target gene *FBLN5* binds integrins to modulate differentiation in the hair shaft and HF development [76]. *MSTRG.52443.14* target gene *CDKN1A* and *PNPLA4.* CDKIs are potent inhibitors of cell cycle progression that are transcriptionally up-regulated in the bulge at all phases or at the start of HF quiescence [77]. *CDKN1A* may play important role in antagonizing the progression of HF development to cause cell cycle arrest at the catagen phase. *PNPLA4* may influence epidermal homeostasis via transcription factor ligands such as retinoid and triglyceride [78]. *MSTRG.45465.3* target gene *GAREM1* plays important role in the regulation of cell proliferation. The *GAREM1* is functionally important in ERK/MAPK signaling pathway and it is implicated in Y-27632 to induce proliferation of different stages ligament stem cells [79, 80]. It also regulates keratinocyte proliferation and differentiation [79]. *MSTRG.840.3* target gene *GSK3β* and *LOC108638286*. The downregulation of *GSK3β* in this study, may be as a result of activation of WNT signaling. *GSK-3β* downregulation may inhibit the formation of the axin complex and indirectly elevates the level of β -catenin. Its inhibitors increase β-catenin levels in HF morphogenesis and SC differentiation in the hair cycle, hence initiating the transcription of a number of downstream genes [81, 82]. It has been shown that plucked human hair contained cells with high *GSK-3β* levels [81]. The downregulation of *LOC108638286* (*KRTAP9–2)* may affect keratinization, as well as HF development and cycling [83]. Keratin-associated proteins (KRTAPs) aid in the development of rigid hair shaft by forming large disulfide bonds with keratin intermediate filament proteins [50]. A recent study revealed *(KRTAP9–2)* expression in all phases of the HF cycle. *MSTRG.23343.1* target gene *MCC* is involved in the negative regulation of cell cycle progression. It serves as a candidate colorectal tumor suppressor gene and suppresses cell proliferation and WNT/*β*-catenin pathway in colorectal cancer cells [84]. Its role in HF development and growth needs further studies. *MSTRG.32111.1* target *BIRC3* and *YAP1*. *BIRC3* regulates apoptosis, cell proliferation, cell invasion and metastasis. *YAP1* is a transcriptional coactivator that regulates Hippo signaling pathway to control homeostasis, proliferation and differentiation [85]. It has shown to regulate SC activation and development cycling as well as regeneration of HFs in adult mice [85]. A previous study in mouse showed that *K14* regulates early differentiation and proliferation through interactions with 14–3-3 adaptor proteins and *YAP1*. More studies should be done in the future to identify the specific interactions among certain genes identified in this study. Protein interaction network for DE lncRNA target genes demonstrated that *ENSBTAG00000025441 (HSPA1A)* and *ENSBTAG00000004912 (ANKRD17)* were key regulators of the cell cycle and innate immune defense.

## Conclusion

In this study, we used RNA-Seq technique to screen and analyze the roles of DE mRNA and lncRNA among different cycles (anagen, catagen and telogen) in Jiangnan cashmere goat SHF. We identified DEGs in SHF potentially related to hair development and cycling. The DEGs included *CHST11, SH3BP4, FBLN5, CDKN1A, PNPLA4, GAREM1, GSK3β, KRTAP9–2, BIRC3, GLYATL2, KRT4, SLC1A2, FA2H, KRT2, KRT39, FAM167A* and *EGFL6* and they were related to cell cycle, cell differentiation, proliferation, apoptosis, immune response, keratinization, proliferation, development, transcription regulation and cell growth. The results of this study could be used to facilitate further studies of the detailed molecular mechanisms in hair development and also serve as a foundation for future studies to potentially manipulate cashmere traits.

## Data Availability

The datasets presented in this study can be found in online repositories. The name of the repository/repositories and accession numbers can be found at Sequence Read Archive (SRA) repository, PRJNA778726.
